# Evolutionary and phylogenetic analyses of 11 *Cerasus* species based on the complete chloroplast genome

**DOI:** 10.3389/fpls.2023.1070600

**Published:** 2023-03-03

**Authors:** Tian Wan, Bai-xue Qiao, Jing Zhou, Ke-sen Shao, Liu-yi Pan, Feng An, Xu-sheng He, Tao Liu, Ping-ke Li, Yu-liang Cai

**Affiliations:** ^1^ College of Horticulture, Northwest Agriculture & Forestry University, Yangling, China; ^2^ College of Natural Resources and Environment, Northwest Agriculture & Forestry University, Yangling, China; ^3^ Center of Experimental Station, Northwest Agriculture & Forestry University, Yangling, China

**Keywords:** *Prunus*, plastome, genomic variation, repeat sequence, protein-coding gene, phylogeny

## Abstract

The subgenus *Cerasus*, one of the most important groups in the genus *Prunus sensu lato*, comprises over 100 species; however, the taxonomic classification and phylogenetic relationships of *Cerasus* remain controversial. Therefore, it is necessary to reconstruct the phylogenetic tree for known *Cerasus* species. Here, we report the chloroplast (cp) genome sequences of 11 *Cerasus* species to provide insight into evolution of the plastome. The cp genomes of the 11 *Cerasus* species (157,571–158,830 bp) displayed a typical quadripartite circular structure. The plastomes contain 115 unique genes, including 80 protein-coding genes, four ribosomal RNAs, and 31 transfer RNAs. Twenty genes were found to be duplicated in inverted repeats as well as at the boundary. The conserved non-coding sequences showed significant divergence compared with the coding regions. We found 12 genes and 14 intergenic regions with higher nucleotide diversity and more polymorphic sites, including *matK*, *rps16*, *rbcL*, *rps16*-*trnQ*, *petN*-*psbM*, and *trnL*-*trnF*. During cp plastome evolution, the codon profile has been strongly biased toward the use of A/T at the third base, and leucine and isoleucine codons appear the most frequently. We identified strong purifying selection on the *rpoA*, *cemA*, *atpA*, and *petB* genes; whereas *ccsA*, *rps19*, *matK*, *rpoC2*, *ycf2* and *ndhI* showed a signature of possible positive selection during the course of *Cerasus* evolution. In addition, we further analyzed the phylogenetic relationships of these species with 57 other congenic related species.Through reconstructing the *Cerasus* phylogeny tree, we found that true cherry is similar to the flora of China forming a distinct group, from which *P. mahaleb* was separated as an independent subclade. *Microcerasus* was genetically closer to *Amygdalus*, *Armeniaca*, and *Prunus* (*sensu stricto*) than to members of true cherry, whereas *P. japonica* and *P. tomentosa* were most closely related to *P. triloba* and *P. pedunculata*. However, *P. tianshanica* formed a clade with *P. cerasus*, *P. fruticosa*, *P. cerasus* × *P. canescens* ‘Gisela 6’, and *P. avium* as a true cherry group. These results provide new insights into the plastome evolution of *Cerasus*, along with potential molecular markers and candidate DNA barcodes for further phylogenetic and phylogeographic analyses of *Cerasus* species.

## 1 Introduction

Rosaceae is a large family that includes most economically important fruits species in temperate zones, such as *Prunus*, *Armeniaca*, *Amygdalus*, *Pyrus*, *Malus* and *Crataegus* species (Zarei et al., 2017; Sevindik et al., 2020). And the plant subgenus *Cerasus* is considered one of the most important groups in the genus *Prunus (P.) sensu lato*, comprising over 100 species, which are naturally distributed in temperate Asia, Europe, North America, China, Japan, and Korea (Chin et al., 2014; Zhang et al., 2021). In China, there are roughly 45 species of *Cerasus*, 35 of which are considered to be endemic according to the Flora of China project (Yü et al., 1986). However, the taxonomic classification and phylogenetic relationships among species in the subgenus *Cerasus* or genus *Prunus sensu lato* have been controversial, with no unification reached to date (Potter et al., 2007; Chin et al., 2014; Liang et al., 2018; Zhang et al., 2021). For example, *P. tomentosa*, *P. tianshanica*, *P. japonica*, *P. humilis*, *P. dictyoneura*, *P. glandulosa*, *P. pogonostyla*, *P. jacquemontii*, *P. prostrata*, and *P. pumila* were classified in a single group, the “dwarf cherry” (*Microcerasus*), which was identified as a section of subgenus *Cerasus* according to Yü et al. (1986) and Webster and Looney (1996). However, in previous phylogenetic studies, *Microcerasus* species corresponded with *Amygdalus*, *Armeniaca*, or *Prunus* species (Bortiri et al., 2001; Shaw and Small, 2004; Chen et al., 2018; Zhang et al., 2021). Therefore, it is necessary to reconstruct the phylogenetic tree for *Cerasus* species.

The origin of the chloroplast (cp) can be traced back more than one billion years (Wang et al., 2021; Ravi et al., 2007). In land plants, the cp genome has a relatively conserved quadripartite structure, with conserved sequences in the range of 120–218 kb (Daniell et al., 2016) encoding approximately 100–130 genes (Palmer, 1985; Ravi et al., 2007; Wicke et al., 2011; Daniell et al., 2016; Wang et al., 2021). The cp structure comprises one large single-copy (LSC) region, one small single-copy (SSC) region, and two copies of an inverted repeat (IR) (Sugiura, 1992; Ravi et al., 2007). The significant developmental impact and limited coding potential of the cp genome, combined with maternal inheritance of this organelle provide tangible, causal approaches to understanding plant evolution, diversity, and phylogenic relationships (Ravi et al., 2007; Moore et al., 2010; Greiner et al., 2011). Recent studies have demonstrated that cp genome sequences offer remarkable resolution for analyzing phylogenetic relationships at various taxonomic levels, and can further provide evidence to explain effects of geography and climate oscillations on genetic divergence (Ivanova et al., 2017; Xue et al., 2019; Xue et al., 2021; Zhang et al., 2021; Dunning et al., 2022; Wang et al., 2022). Although cp genome sequences of some *Cerasus* species have been published (Chen et al., 2018; Feng et al., 2018; Zhang et al., 2021; Li et al., 2022), there is still a lack of information to enable comprehensive analysis of the interspecific relationships of the subgenus *Cerasus* and the relationship between *Cerasus* and *Prunus sensu lato*. Comparison of the cp genomes of the 11 *Cerasus* species can help to better understand evolution of the *Cerasus* genome and enable more profound analysis of the phylogenetic relationships in the genus *Prunus*, offering valuable insights.

In this study, we performed a comparative analysis of 11 complete *Cerasus* cp genomes to explore the features and structural differentiation of sequences among species. Furthermore, we reconstructed a phylogenetic tree using the newly obtained cp chloroplast sequences and published sequences to explore the genetic relationships among subgenus *Cerasus*, *Prunus* (*sensu stricto*), *Amygdalus*, and *Armeniaca*. Our study objectives were to: (1) gain insight into plastome structure features, (2) inform an improved understanding of cp genome evolution, and (3) further delineate the taxonomic status of *Cerasus*.

## 2 Materials and methods

### 2.1 Plant materials, sequencing, cp genome assembly, and annotation

Fresh and healthy leaves were collected from adult plants of 11 *Cerasus* species (**Table 1**). All samples were immediately frozen in liquid nitrogen and stored at −80°C until analysis. Total genomic DNA was extracted from 100 mg of fresh leaves using a modified CTAB method (Murray and Thompson, 1980). DNA libraries were prepared and sequenced on an Illumina NovaSeq 6000 platform (Illumina, San Diego, CA, USA) with paired-end 150-bp sequencing reads; only reads with a Q30 quality score greater than 80% were retained for analysis.

**Table 1:** Sampling information for the *Cerasus* species.

No.	Species	Origin	Sampling sites, longitude, latitude	GenBank number
1	*P. avium* (wild)	Hungary	Mei County, Shaanxi, China E 107.9908°, N 34.1123°	OP598110
2	*P. cerasus*	Hungary	Mei County, Shaanxi, China E 107.9908°, N 34.1123°	MW477432
3	*P. cerasus × P. canescens ‘*Gisela *6’*	Germany	Qishan County, Shaanxi, China E 107.6371°, N 34.3749°	MW477433
4	*P. fruticosa*	Hungary	Mei County, Shaanxi, China E 107.9908°, N 34.1123°	MW477434
5	*P. japonica*	Shanxi, China	Hongdong, Shanxi, China E 111.8234°, N 36.4298°	OP598111
6	*P. mahaleb*	Hungary	Mei County, Shaanxi, China E 107.9908°, N 34.1123°	MW477435
7	*P. serrula*	Yunnan, China	Mei County, Shaanxi, China E 107.9908°, N 34.1123°	MW477436
8	*P. serrulata*	Shandong, China	RiZhao, Shandong, China E 119.2087°, N 35.7501°	OP611546
9	*P. tianshanica*	Xinjiang, China	Yili, Xinjiang, China E 81.2771°, N 43.9094°	OP598112
10	*P. tomentosa*	Shaanxi, China	Taibai, Shaanxi, China E 107.5947°, N 34.0533°	MW477437
11	*P. trichostoma*	Xizang, China	Nyingchi, Xizang, China E 94.6609°, N 29.6340°	OP598113

The cp genome assembly of *Cerasus* species was obtained by a baiting and iterative mapping approach (Hahn et al., 2013). The complete cp genome of *P. persica* (HQ336405) was downloaded from the National Center for Biotechnology Information (NCBI) database as a reference genome. Geneious Prime v2022.0.2 (https://www.geneious.com/) (Kearse et al., 2012) was used for sequence correction. The 11 *Cerasus* species were annotated by Geneious Prime v2022.0.2, using *P. pseudocerasus* (NC030599) and *P. persica* (HQ336405) as reference sequences, and were annotated using GeSeq (https://chlorobox.mpimp-golm.mpg.de/geseq.html) with no reference sequence. Manual editing of annotated and non-annotated portions, including exons and introns, was then performed. The transfer RNA (tRNA) sequences were confirmed using tRNAscan-SE 2.0 (Chan et al., 2021). All annotations were checked against the reference genomes (NC030599, NC054254, and MZ145044). Genome maps were drawn using OrganellarGenomeDRAW (OGDRAW) (Greiner et al., 2019).

### 2.2 Complete cp genome comparison

Plastome structures among *Cerasus* species, apple, pear, and grape were compared by the mVISTA percent identity plot in Shuffle-LAGAN mode to reveal the major genomic variations located in LSC and SSC regions (Brudno et al., 2003; Frazer et al., 2004). Subsequently, nucleotide diversity (*Pi*) and polymorphic sites (*S*) of single-copy genes and intergenic regions (IGRs) were estimated for the 11 species and *P. pseudocerasus* (NC030599) by DnaSP v.6 (Rozas et al., 2017). The plastome genetic architecture of the 11 *Cerasus* species, 24 *Cerasus* species available in NCBI, and six other species for the LSC/IR and SSC/IR boundaries were analyzed by Irscope (Amiryousefi et al., 2018).

### 2.3 Repeat sequences analysis

Simple sequence repeats (SSRs) were examined by the Perl script MicroSAtellite (MISA) (Beier et al., 2017) with the following parameter settings: motif size of 1–6 nucleotides; and minimum repeat unit of 10 for mononucleotides, 6 for dinucleotides, and 5 for tri-, tetra-, penta-, and hexa-nucleotides. Non-overlapping repeat sequences were identified by REPuter (Kurtz et al., 2001) (repeat unit length minimum ≥ 25 bp, Hamming distance = 3). Four matches of repeats were classified, namely forward, reverse, complement, and palindromic matches. The online program Tandem Repeats Finder (http://tandem.bu.edu/trf/trf.html) was used to find the tandem repeat sequences of at least 10 bp in length. The alignment parameters for match, mismatch, and indels were set to 2, 7, and 7, respectively.

### 2.4 Codon usage bias and gene selective pressure analysis

For identification of codon usage patterns, all coding sequences (CDSs) greater than 350 nucleotides in length were extracted from the cp genome of *Cerasus*, as described previously (Morton, 1998). The filtered CDSs were subsequently used for the estimation of codon usage using CodonW v.9.1.2 and the codon usage patterns were analyzed by GraphPad Prism v.8.0. In addition to the overall codon usage, we further tabulated codon usage measures such as the effective number of codons (Nc) and GC frequency at the third synonymous position (GC3s). To investigate the selective pressure on plastome protein-coding genes between two species, non-synonymous (Ka) and synonymous (Ks) substitution values were calculated by KaKs_Calculator 2.0 (Wang et al., 2010), with the following settings: genetic code table = 11 (bacterial and plant plastid codes) and the Yang-Nielsen algorithm (YN) calculation method (Ivanova et al., 2017).

### 2.5 Phylogenetic relationship

To reconstruct the phylogenetic relationships and verify the phylogenetic position of the subgenus *Cerasus* in *Prunus sensu lato*, 45 *Cerasus* cp genome sequences, including 34 published sequences downloaded from NCBI and the 11 sequences obtained in this study, were analyzed along with 23 *P.sensu lato* complete cp genome sequences. *Malus baccata*, *Malus micromalus*, *Pyrus communis*, *Pyrus ussuriensis*, *Vitis amurensis*, *V. amurensis*, and *Ziziphus jujuba* were regarded as outgroups. Because the different regions of cp genome differed in the molecular evolutionary rates (Zhang et al., 2017), phylogenetic relationship analyses were performed using 6 datasets that are the complete cp genome sequences, LSC regions, SSC regions, two IR regions, common CDS and IGRs. Sequences were aligned by MAFFT using Geneious Prime v2022.0.2 (Kearse et al., 2012). The phylogenetic tree was constructed with the program Mrbayes (Huelsenbeck and Ronquist, 2001) of Geneious Prime and Maximum likelihood (ML) method of MEGA v11(Kumar et al., 2016), Mrbayes analysis used the following Markov chain Monte Carlo simulation settings: chain length = 1,100,000, subsampling frequency = 200, heated chains = 4, burn-in length = 100,000, heated chain temp = 0.2, and random seed = 170. ML analyses used the General Time Reversible +Gamma Distributed + Nearest-Neighbor-Interchange model with 1000 bootstrap replicates. The tree was visualized with Interactive Tree Of Life (iTOL) v5 software (Letunic and Bork, 2021) and manually edited where necessary.

## 3 Results

### 3.1 Cp genome structure of *Cerasus* species

**Figure 1:**
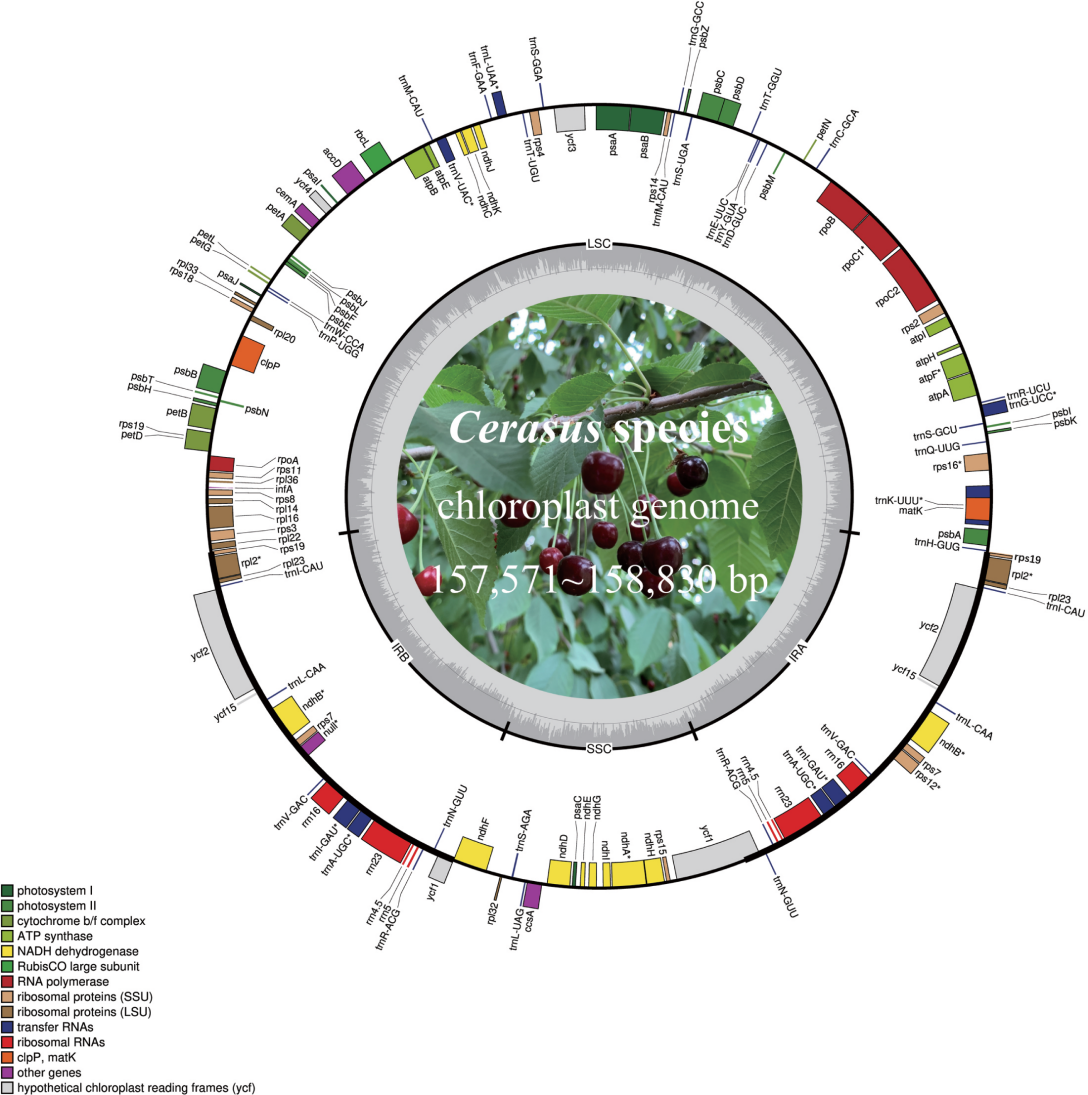
Chloroplast genome map of *Cerasus* species. Genes on the inside of the large circle are transcribed clockwise and those on the outside are transcribed counterclockwise. The genes are color-coded based on their functions. The dashed area represents the GC composition of the chloroplast genome. LSC, large single copy region; IR, inverted repeat; SSC, small single copy region.

The *Cerasus* cp genome displays a typical quadripartite circular structure (**Figure 1**) containing one LSC, one SSC, and two IR (IRB and IRA) regions, as determined by OGDRAW (Greiner et al., 2019). The plastome size of the 11 *Cerasus* species ranged from 157,571 bp (*P. tomentosa*) to 158,830 bp (*P. tianshanica*). The average coverage depth ranged from 421.8× to 9410× (**Table S1**; **Figure S1**). The GC content of the *Cerasus* cp genome was very similar among the 11 species (36.5–36.8%) with an average of 36.7% (**Table S1**).

*Cerasus* plastomes contained the same set of 115 unique genes, including 80 protein-coding genes, 4 ribosomal RNAs (rRNAs), and 31 tRNAs (**Table 2**). Twenty genes are duplicated in IRs or at the boundaries, including nine protein-coding genes (*rpl2*, *rpl23*, *rps7*, *rps12*, *rps19*, *ndhB*, *ycf1*, *ycf2*, and *ycf15*), four rRNA genes (*rrn4.5*, *rrn5*, *rrn16*, and *rrn23*), and seven tRNA genes (*trnA-UGC*, *trnI-CAU*, *trnI-GAU*, *trnL-CAA*, *trnN-GUU*, *trnR-ACG*, and *trnV-GAC*). In the cp genome of *P. tianshanica*, an insertion sequence was identified that split *ycf2* in IRA into two segments (**Figure S2A**). There are 18 different intron-containing genes (**Table 2**), including 10 protein-coding genes (*atpF*, *ndhA*, *ndhB*, *rpl2*, *rpl16*, *rps12*, *rps16*, *rpoC1*, *petB*, and *petD*) and six tRNA-coding genes (*trnA-UGC*, *trnG-UCC*, *trnI-GAU*, *trnK-UUU*, *trnL-UAA*, and *trnV-UAC*). Among these, *trnK-UUU* has the largest intron (2428–2539 bp) with *matK* located within it. Two protein-coding genes (*clpP* and *ycf3*) contain two introns.

**Table 2:** Gene types and functional classification of the *Cerasus* chloroplast genome.

Category	Gene group	Gene symbol				
Self-replication	Ribosomal RNA genes	*rrn4.5* ^a^	*rrn5* ^a^	*rrn16* ^a^	*rrn23* ^a^	
	Transfer RNA genes	*trnA-UGC* ^a;b^	*trnC-GCA*	*trnD-GUC*	*trnE-UUC*	*trnF-GAA*
		*trnfM-CAU*	*trnG-GCC*	*trnG-UCC* ^b^	*trnH-GUG*	*trnI-CAU* ^a^
		*trnI-GAU* ^a;b^	*trnK-UUU^b^*	*trnL-CAA* ^a;b^	*trnL-UAA* ^b^	*trnL-UAG*
		*trnM-CAU*	*trnN-GUU^a^*	*trnP-UGG*	*trnQ-UUG*	*trnR-ACG* ^a^
		*trnR-UCU*	*trnS-AGA*	*trnS-GCU*	*trnS-GGA*	*trnS-UGA*
		*trnT-GGU*	*trnT-UGU*	*trnV-GAC^a^*	*trnV-UAC* ^b^	*trnW-CCA*
		*trnY-GUA*				
	Small subunit of ribosome	*rps2*	*rps3*	*rps4*	*rps7* ^a^	*rps8*
		*rps11*	*rps12* ^a;b^	*rps14*	*rps15*	*rps16* ^b^
		*rps18*	*rps19* ^d^	*rps36*		
	Large subunit of ribosome	*rpl2* ^a;b^	*rpl14*	*rpl16* ^b^	*rpl20*	*rpl22*
		*rpl23* ^a^	*rpl32*	*rpl33*	*rpl36*	
	DNA-dependent RNA polymerase	*rpoA*	*rpoB*	*rpoC1* ^b^	*rpoC2*	
Photosynthesis	Subunits of photosystem I	*psaA*	*psaB*	*psaC*	*psaI*	*psaJ*
	Subunits of photosystem II	*psbA*	*psbB*	*psbC*	*psbD*	*psbE*
		*psbF*	*psbH*	*psbI*	*psbJ*	*psbK*
		*psbL*	*psbM*	*psbN*	*psbT*	*psbZ*
	Subunits of cytochrome	*petA*	*petB^b^*	*petD^b^*	*petG*	*petL*
		*petN*				
	Subunits of ATP synthase	*atpA*	*atpB*	*atpE*	*atpF^b^*	*atpH*
		*atpI*				
	Large subunit of RuBisCO	*rbcL*				
	Subunits of NADH dehydrogenase	*ndhA* ^b^	*ndhB* ^a;b^	*ndhC*	*ndhD*	*ndhE*
		*ndhF*	*ndhG*	*ndhH*	*ndhI*	*ndhJ*
		*ndhK*				
Other genes	Maturase	*matK*				
	Translational initiation factor	*infA*				
	Envelope membrane protein	*cemA*				
	Subunit of acetyl-CoA	*accD*				
	C-type cytochrome synthesis gene	*ccsA*				
	Protease	*clpP* ^c^				
	Proteins of unknown function	*ycf1* ^a;d^	*ycf2* ^a^	*ycf4*	*ycf3* ^c^	*ycf15* ^a^

^a^Two gene copies in inverted repeats; ^b^gene containing a single intron; ^c^gene containing two introns; ^d^gene divided into two independent transcription units.

In *Cerasus* plastomes, the protein-coding gene *rps19* is located on the boundary of the LSC and IR regions, except for *P. avium* in which only the *rps19* fragment is on IRA. Comparison showed significant differences of *rps19* gene sequences among the *Cerasus* species (**Figure S2B**). The *ycf1* gene was located on the boundary of the SSC and IRA regions. *ycf* genes were identified as hypothetical cp reading frames, and small fragments of truncated *ycf* genes were detected in IRA (*ycf15*: 126 bp and 129 bp, respectively), with only partial *ycf1* identified in the IRB region. The remaining *ycf* genes were detected at the complete gene size. *ycf2* is a large functional gene encoding 2277 amino acids in cp IR regions. The *ycf2* gene in *P. tianshanica* was 6876 bp, with one inserted fragment of 42 bp located at the 900-bp position (**Figure S2A**). In *Cerasus*, a high level of similarity was restricted to the IRs, and major differences originated from the LSC and SSC regions. The gene *infA*, which is a translation-related gene, was identified as a pseudogene.

### 3.2 Complete cp genome sequence comparison of 11 *Cerasus* species

**Figure 2:**
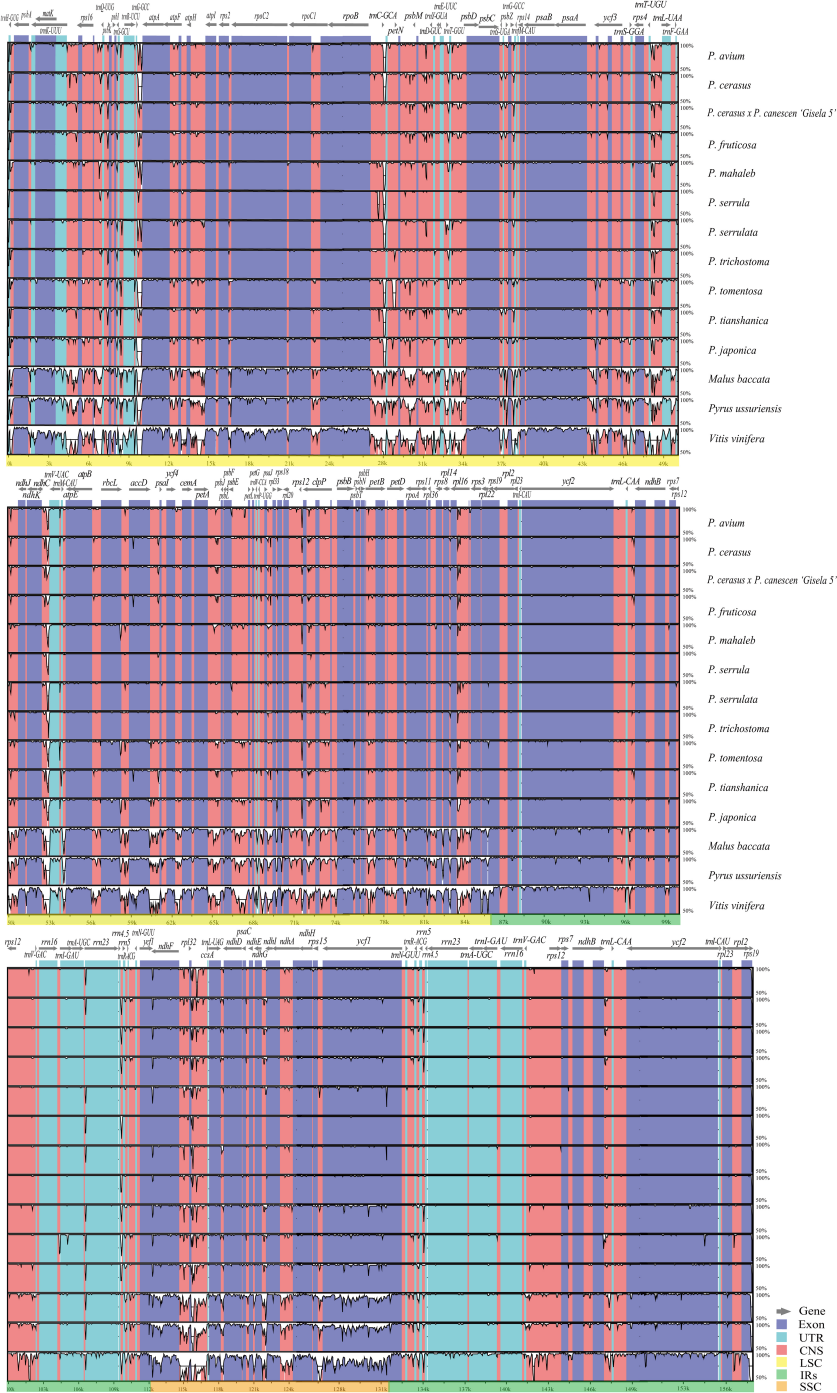
Chloroplast genome sequence comparison of 11 *Cerasus* species, apple, pear, and grape based on mVISTA. The similarity graphical information portrays sequence identity with *Prunus pseudocerasus* (NC030599) as reference. A cut-off of 50% identity is used for the plots. In each plot, the Y-axis represents percent identity (50–100%).

The mVISTA (Frazer et al., 2004) analysis showed the overall sequence identity, divergent regions, and visualization of the aligned cp genome sequences in *Cerasus*. The LSC and SSC regions were clearly more divergent than the IRs (**Figure 2**). The conserved non-coding sequences (CNSs) showed significantly more divergence than the coding regions (**Figure 2** and **Table S2**), indicating that the CDSs are much more conserved than the CNSs. Furthermore, the mean value of *Pi* in IRs (0.00157) was lower than that of the LSC (0.00719) or SSC (0.01041) regions, which demonstrated that the IR regions have fewer mutations and are thus more strongly conserved. Among 135 plastid genes, only 23 genes showed higher nucleotide diversity (*Pi* > 0.003), with *Pi* values ranging from 0.00307 (*rpoC2*) to 0.00777 (*rps15*), and 12 genes (*trnK-UUU*, *matK*, *rps16*, *trnG-UCC*, *rpoC2*, *rbcL*, *accD*, *clpP*, *rpl16*, *ndhF*, *ndhA*, and *ycf1*) had a relatively higher number of polymorphic sites (*S* > 10) (**Table S2A**; **Figure S3**). However, 73 IGRs had *Pi* > 0.003; the 10 most polymorphic IGRs in ascending order were *rps19*-*trnG-GUU* (*Pi* = 0.06926), *trnR-UCU*-*atpA*, *ndhC*-*trnV-UAC*, *ccsA*-*ndhD*, *psbl*-*trnS-GCU*, *psbC*-*trnS-UGA*, *rpl32*-*trnS-AGA*, *rpl33*-*rps18*, *trnW-CCA*-*trnP-UGG*, and *psbZ*-*trnG-GCC* (*Pi* = 0.01334). Moreover, there were 14 IGRs with *Pi* > 0.01 and *S* > 10 (**Table S2B**; **Figure S3**). We further analyzed the sequence divergence patterns among all cp genomes. Finally, 506 single nucleotide polymorphisms (SNPs), 59 nucleotide substitution (NS) loci, and 1898 indel loci were identified through the nucleotide alignment (**Table S3**).

**Figure 3:**
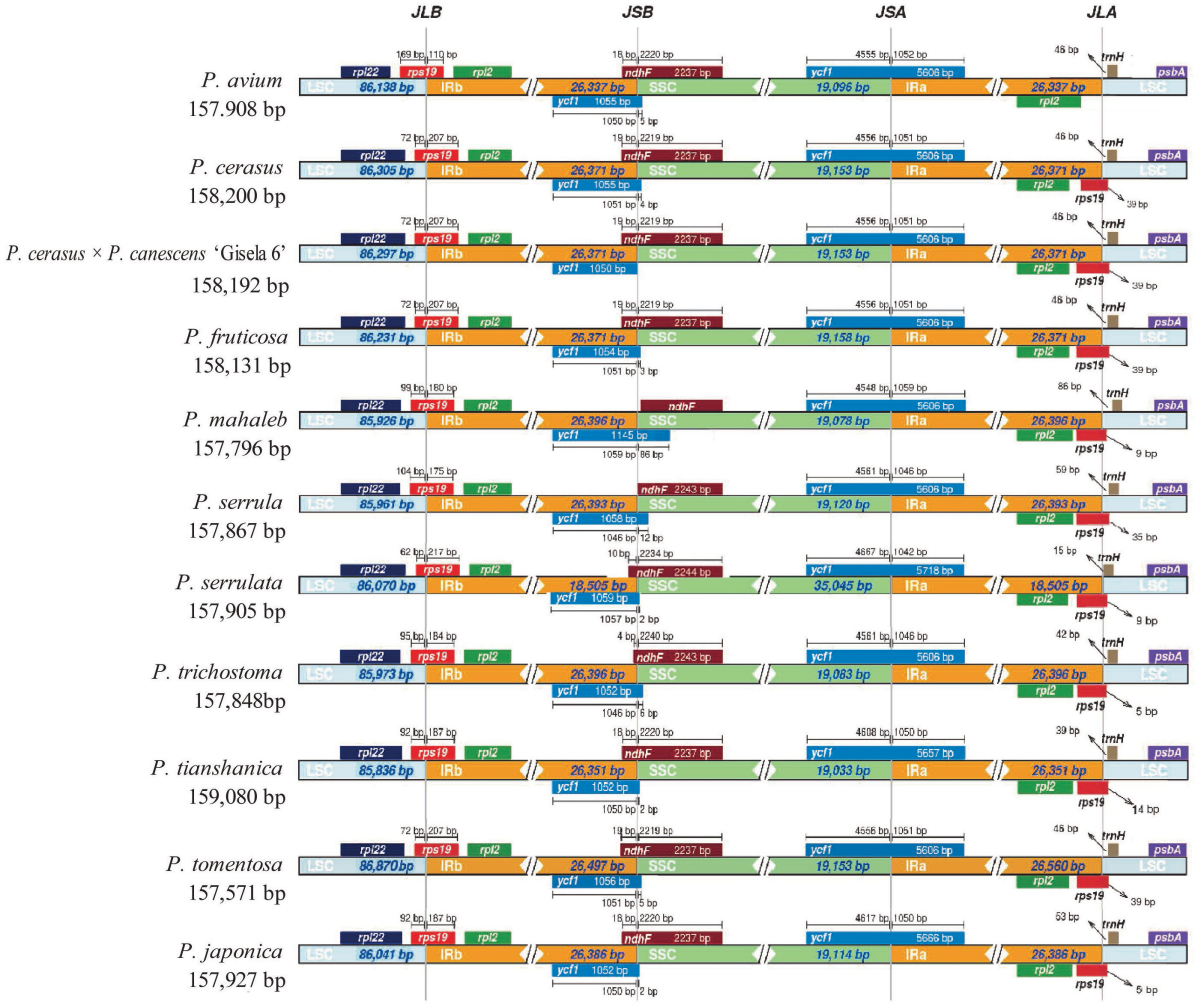
Comparison of the inverted repeat (IR)/large single copy (LSC) boundaries and IR/short single copy (SSC) boundaries among chloroplast genomes of 11 *Cerasus* species. JLA and JLB indicate the junction sites between the SSC and the two IRs (IRA and IRB); JSA and JSB denote the junction sites between the SSC and the two IRs.

Comparing the IR/LSC and IR/SSC boundaries of the 11 cp genomes of *Cerasus* species revealed the contraction and expansion of IRs with minimal variation at the boundaries. The boundary region of IRA/SSC appears to be relatively stable (**Figures 3**, **S3**). That is, the boundary gene *ycf1* showed high conservation among *Cerasus* species with a length of 5606 bp for the majority of the *Cerasus* species (7/11) analyzed. In *P. serrulata*, *P. tomentosa*, and *P. japonica*, *ycf1* had greater extension than found in the other species to different degrees (**Figure 3**). Both the *ycf1* pseudogene and *ndhF* gene were at the IRB/SSC borders, which partially overlapped in the cp genomes of *Cerasus* species. The IRB/LSC junction was largely located in *rps19*, close to *rpl22* and *rpl2*, and except in *P. avium*, extension of the LSC resulted in larger contraction of IRB toward the *rpl22* direction. Concerning the IRA/LSC boundary, the junction site was the *rps19* gene in all *Cerasus* species, except for *P. avium* in which *rps19* showed a contraction in IRA regions, being 48 bp away from the boundary. In addition, *trnH* was consistently observed in all plastomes, which was located 5–86 bp away from the border.

### 3.3 Repeat sequence analysis

**Figure 4:**
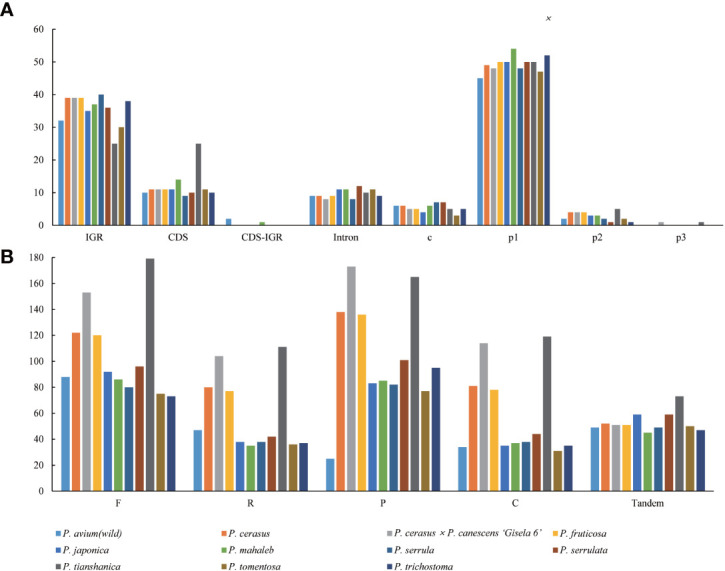
Repetitive motif abundance in 11 *Cerasus* species computed by REPuter and Tandem Repeats Finder. **(A)** Distribution and types of simple sequence repeats in the eleven chloroplast genomes. **(B)** Number of repeat types in the eleven chloroplast genomes.

The types and distribution of SSRs were analyzed in the cp genomes of the 11 *Cerasus* species. A total of 634 SSRs were identified by MISA, 61.51% of which were distributed in the IGR and 133 of which were found in CDSs (**Figure 4A**; **Table S4A**). Except for *P. tianshanica*, the SSRs were mainly enriched in the IGRs and CDSs, both accounting for 41.67% of all SSRs. For the other *Cerasus* species, SSRs were more abundant in IGRs than in other regions (**Figure 4A**; **Table S4A**). Moreover, four types of SSRs were identified: mono-, di-, and tri-nucleotide, and complex repeats. Mononucleotide repeats were the most common, accounting for 85.65% of the total (n =543, range 45–54; **Table S4A**). By contrast, only 31 dinucleotide repeats were identified (ranging from 1 to 5), accounting for 4.89% of the total, and only two trinucleotide repeats were found, in *P. cerasus* × *P. canescens* ‘Gisela 6’ and *P. tianshanica*, respectively. No other polynucleotides were detected. In addition, the composition of the mononucleotide repeats was mostly A/T, with C and G repeats accounting for less than 6% of these repeats (**Table S4A**), and all dinucleotide repeats were composed of AT/TA. For the non-overlapping repeats, there were 1164 forward repeats, 645 reverse repeats, 1160 palindromic repeats, 646 complement repeats, and 585 tandem repeats identified using REPuter (Kurtz et al., 2001) and Tandem Repeats Finder for the *Cerasus* plastomes (**Table S4B**). Forward repeats were the most abundant (n = 73–179), followed by palindromic repeats (n = 25–173). Tandem repeats were the least abundant repeat type, ranging from 45 in *P. mahaleb* to 73 in *P. tianshanica* within 5–249 bp (**Table S4**). Dispersed repeats were more common in *P. cerasus* × *P. canescens* ‘Gisela 6’ and *P. tianshanica* than in the other species (**Figure 4B** and **Table S4B**).

### 3.4 Codon usage bias and gene selective pressure analysis

**Figure 5:**
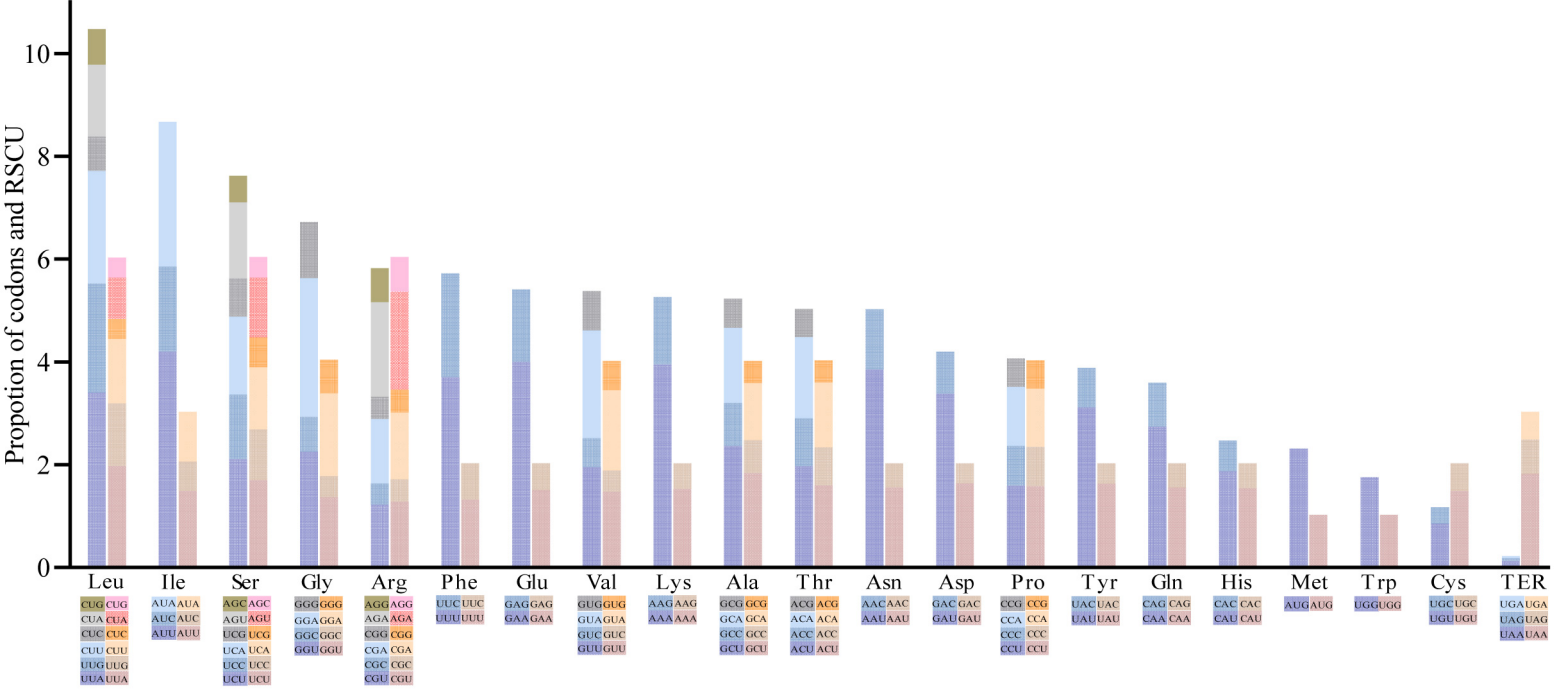
Codon content of 20 amino acids and termination codons in protein-coding genes of *Cerasus* plastomes. The left columns of the histogram present codon usage and the right columns denote the relative synonymous codon usage (RSCU) values. Colors correspond to codons listed underneath the columns.

A total of 49 *Cerasus* genes were selected based on the 350-bp length threshold for codon bias identification. Codon usage measures such as Nc; silent T, C, A, and G at the 3^rd^ codon position (T3s, C3s, A3s and G3s, respectively); total number of amino acids (L_aa); aromaticity; and grand average of hydropathicity (GRAVY) were estimated (**Tables S4A**, **S5**). The number of synonymous codons (L_sym) was approximately 49.7 and the range of L_aa, except for the termination codon (TER), was from 24,034 in *P. cerasus* × *P. canescens* ‘Gisela 6’ to 24,421 in *P. serrulata*, with a relative synonymous codon usage (RSCU) value ranging from 0.38 (CUC) to 1.96 (UUA) (**Tables S5B, C**; **Figures S5**, **5**). The average Nc was 49.74 for CDSs of the 11 *Cerasus* species, and the T3s, C3s, A3s, and G3s was 0.4674, 0.1718, 0.4356, and 0.1817, respectively. The mean of GC3s was 0.269 and the GC content was 0.376 (**Table S5A**). In addition, leucine and isoleucine were the most common codons (**Figure 5**). Methionine (AUG) and tryptophan (UGG) were each encoded by only one codon, and showed no codon bias (**Figure 5**). Codon usage was biased toward A and T at the third codon position. Almost all A/U-ending codons had RSCU values larger than 1.0, except for Ile-AUA, Leu-CUA, and TER-UGA, whereas all C/G-ending codons had RSCU values ≤ 1, except for Leu-UUG (**Figure S5**; **Table S5C**).

The synonymous (Ks) and non-synonymous (Ka) nucleotide substitution patterns are very important markers in gene evolution studies (Kimura, 1989). In all protein-coding genes of *Cerasus*, *ndhB*, *petL*, *psbH*, *rpl33*, *rps18*, and *ycf15* had no nonsynonymous rate change, and 20 genes (*psaJ*, *psbA*, *atpE ndhE*, *ndhJ*, *psaC*, *petD*, *ycf3*, *ycf4*, three *rpl* genes, three *rps* genes, and five *psb* genes) had no synonymous rate change. Fourteen genes showed neither substitution, including *rpl23*, *rps7*, *rps8*, *rps12*, *petG*, *petN*, *psaI*, *psbI*, *psbJ*, *psbK*, *psbL*, *psbM*, *psbN*, and *psbT*. **Table S6** lists the genes with both Ka and Ka substitutions. A Ka/Ks ratio < 1, especially less than 0.5, indicates purifying selection; Ka/Ks > 1 indicates likely positive selection; and Ka/Ks values close to 1 show neutral evolution, or relaxed selection (Kimura, 1989; Ivanova et al., 2017). The average Ka/Ks ratio analyzed in the 11 genomes was 0.3158 for 34 protein-coding genes, which were not region-specific. Most protein-coding genes have undergone purifying selection: 33 coding genes showed Ka/Ks < 1, ranging from 0.0301 (*atpA*) to 0.8861 (*ccsA*). *ycf2*_IRA showed Ka/Ks > 1, whereas the ratio for the other *ycf2* gene on IRB was 0.2908. The *ccsA* and *ndh* genes (except for *ndhK* in the LSC region) were located in the SSC region, *ycf1* was located at the boundary of the LSC and IR regions, and other genes existed in the LSC. Moreover, Ka/Ks ratios in the range of 0.5–1 were observed for the genes *petA*, *rps15*, and *ccsA*, with the value of *ccsA* being close to 1, indicating neutral evolution (**Table S6A**; **Figure S6**). The Ka/Ks values of the remaining genes were between 0.04 and 0.50, with *rpoA*, *atpA*, *pet*B, *cemA*, and *rpoC1* showing patterns of strong purifying selection pressure (Ka/Ks < 0.1). For *ycf2*_IRA, *matK*, *rpoC2*, *ccsA*, and *ndhI*, the Ka/Ks ratio was greater than 1 in a few cases between the species (**Table S6B**), especially for *ycf2*_IRA in the comparison of *P. tianshanica* with other species. In addition, we analyzed the Ka/Ks ratios of the 10 cp genomes for comparison with *P. avium*, which reflected the selection pressure for the *P. avium* cp genome (**Figure S7**). The average Ka/Ks values of most genes were less than 0.5, except for *matK*, *rpoC2*, and *ycf2*_IRA. The Ka/Ks value of *matK* was greater than 1 in the comparisons of *P. avium* with *P. japonica*, *P. tomentosa*, and *P. tianshanica* (**Table S6C**).

We found that photosynthesis genes had varying Ka/Ks ratios, which were all less than 1, including one large subunit of the RuBisCO gene *rbcL* (0.1799–0.4581); two subunits of the photosystem II genes *psbB* and *psbC* (0.0643–0.2921); two subunits of the cytochrome genes *petA* and *petB* (0.0561–0.6536); and three subunits of the ATP synthase genes *atpA*, *atpB*, and *atpF* (0.0301–0.3146). The ratios of five subunits of NADH dehydrogenase genes (*ndhA*, *ndhD*, *ndhH*, *ndhG*, *ndhG* and *ndhI*) ranged from 0.04701 to 1.7677, and the Ka/Ks ratio of *ndhI* was greater than 1. Ka/Ks ratios of self-replicating genes were as follows: 0.0566–0.8818 for ribosomal protein small subunit genes (*rps3*, *rps4*, *rps15*, *rps16*), 0.2447–0.496 for ribosomal protein large subunit genes (*rpl16* and *rpl20*), and 0.0327–1.1722 for DNA-dependent RNA polymerase genes (*rpoA*, *rpoB*, *rpoC1*, and *rpoC2*). Among these, only *rpoC2* had Ka/Ks > 1. Among the other genes, *matK*, *ccsA*, *cemA*, *clpP*, *accD*, *ycf1*, and *ycf2*, the Ka/Ks ratios of *matK, ycf2*, and *ccsA* were more than 1 (**Table S6B**).

### 3.5 Phylogenetic analysis

**Figure 6:**
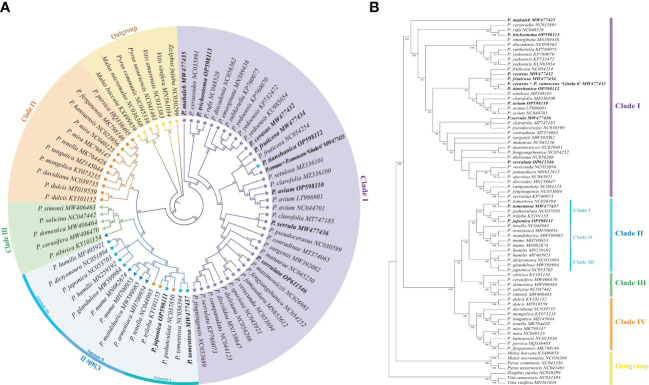
Phylogenetic tree reconstruction of 45 *Cerasus* species and 23 other Prunoideae species based on whole chloroplast genome sequences. **(A)** Phylogenetic tree reconstruction using the program Mrbayes of Geneious Prime v2022.0.2. Numbers above the lines represent the Bayesian inference posterior probability (percent). **(B)** Phylogenetic tree reconstruction using Maximum likelihood method of MEGA v11. Numbers below the lines represent the bootstrap support values.

Organelle genome sequencing plays a key role in deciphering the evolutionary phylogenomics and cladistics of plant species. Phylogenetic relationships of *Cerasus* species were estimated with 6 datasets respectively, using Bayesian inference (BI) and ML methods. The results showed the tree topologies based on LSC, SSC, CDS and IGS datasets is basically consistent with complete plastome, especially using BI method (**Figures S8**–**S12**). Phylogenetic relationships were almost as consistent with BI and ML with the complete cp genome sequences (**Figure 6**). In the ingroup, the subgenera *Cerasus*, *Prunus*, *Armeniaca*, and *Amygdalus* were divided into four major clades (clade I, clade II, clade III, and clade IV) with BI posterior probabilities (BIPP) of 100% and ML bootstrap support (MLBS) of 99–100%. All of the clade I species belonged to subgenus *Cerasus* and all clade IV species belonged to subgenus *Amygdalus*. In clade III, only *P. sibirica* was an *Armeniaca* species, whereas the others belonged to *Prunus*. Among the four clades, clade II showed a relatively more complex composition, including dwarf cherry species (*P. tomentosa*, *P. japonica*, *P. glandulosa*, *P. humilis*, and *P. dictyoneura*) of *Cerasus*, *Amygdalus* (*P. tenella*, *P. pedunculata*, *P. triloba*), and *Armeniaca* (*P. mume*, *P. mandshurica*, and *P. armeniaca*). Through rebuilding the phylogenetic tree, clade II could be further divided into three small clades (BIPP = 99.87–100%; MLBS = 94–100%), named subclade I, subclade II, and subclade III, respectively. All species in subclade III belonged to *Cerasus*, and subclade I was also dominated by *Cerasus* except for *P. triloba*. Conversely, most of the subclade II species belonged to the subgenus *Armeniaca*, except for *P. tenella*. In clade I, *P. mahaleb* formed a separate branch (**Figure 6**). *P. trichostoma* was the closest relative to *P. rufa*. *P. cerasodies*, *P. fruticosa*, *P. cerasus*, *P. tianshanica*, and *P. cerasus* × *P. canescens* ‘Gisela 6’ were divided into the same small clade, which was close to the clade formed by *P. avium*, *P. clarofolia*, and *P. setulasa. P. serrula* was divided into a clade with *P. pseudocerasus* and *P. clarofolia*. *P. serrula* was the closest relative to *P. fengyangshanica*, *P. jamasakura*, *P. dielsiana*, and seven other species. Surprisingly, *P. tomentosa* and *P. japonica* were separated from the majority of *Cerasus* species and were most closely related to *P. triloba* and *P. pedunculata* of the subgenus *Amygdalus* (**Figure 6**).

## 4 Discussion

### 4.1 *Cerasus* cp genome features and genomic variation

The cp genome exhibits maternal inheritance in contrast to the nuclear genome (Palmer, 1985). Because of its evolutionarily conserved structure, sequence length, and constituent genes, the cp genome has been widely used in analyses of genetic variation and phylogeny with moderate base replacement (Palmer, 1985; Ravi et al., 2007; Wicke et al., 2011). In this study, the 11 *Cerasus* cp genomes presented a typical quadripartite structure (LSC, SSC, IRA, and IRB), as reported for other *Prunus* species (Xue et al., 2019; Zhang et al., 2021; Li et al., 2022) and land plants (Wicke et al., 2011; Zhang et al., 2017; Liu et al., 2020). The *Cerasus* cp genome size (157,571–158,830 bp) was similar to that of previously reported *Prunus* species such as *P. pseudocerasus* (157,834 bp), *P. dielsiana* (158,005 bp), *P. clarofolia* (157,899 bp), *P. mira* (158,153 bp), and *P. salicina* (157,916 bp) (Feng et al., 2018; Bao et al., 2019; Xue et al., 2019; Zhao et al., 2019; Li et al., 2022). This indicated that the length of the cp sequence is relatively conserved with only moderate variation among species of the *Prunus* genus.

Comparative analysis of *Cerasus* species showed that the LSC and SSC regions were more divergent than the IR regions (**Figure 2**), whereas the CNSs showed significant divergence (**Figure S3**), consistent with findings for other species (Xue et al., 2019; Liu et al., 2020; Wang et al., 2021). One of the most important factors contributing to the variation in plastome size between species may be the expansion and contraction of IR boundaries (Xue et al., 2019; Zhang et al., 2021). The contraction and expansion of *rps19* and *ndhF* were predicted as the main contributors to the overall variation observed among the *Cerasus* cp genomes, followed by expansion of *ycf1* toward the SSC region (**Figures 3**, **S4**). The three genes at IR boundaries were consistent in *P. mume*, *P*. *salicina*, and *P. armeniaca* (Xue et al., 2019). Significant IR contraction of *rps19* and *ndhF* was observed in the plastomes of some *Cerasus* and other *Prunus* species (Zhang et al., 2021; Wang et al., 2022). In addition, displacement of the *trnH* gene at the IR/LSC locus was detected in the aforementioned studies and the current study. This pattern of these four genes located at IR boundaries was also found in species of other genera, including *Malus* (Zhang et al., 2021; Wang et al., 2022), *Diospyros* (Heinze et al., 2016), and *Morella* (Liu et al., 2017). Therefore, although the IR regions are highly conserved for stabilizing the cp genome structure (**Figure 2**) (Marechal and Brisson, 2010), some changes (especially in *rps19, ndhF*, and *ycf1*) are evident at the IR border areas among *Cerasus* species, in line with reports for other species and genera.

We annotated 115 unique genes in this study (**Table 2**), which is similar to the findings reported for other *Prunus* plastomes with 110–115 unique genes (Xue et al., 2019; Wang et al., 2021; Zhang et al., 2021; Li et al., 2022). There were four rRNA genes detected (4.5S rRNA, 5S rRNA, 16S rRNA, and 23S rRNA), which coincides with reports for land plants (Sugiura, 1992). The differences were mainly reflected in tRNA and protein-coding genes, such as *ycf* genes (Xue et al., 2019; Zhang et al., 2021). The *ycf15* gene was detected in all 11 *Cerasus* cp genomes, but is lacking in some *Prunus* (*sensu lato*) plastomes (Zhang et al., 2021). According to previous reports, angiosperm plastomes harbor approximately 70–88 protein-coding genes (Liu et al., 2020) and 80 unique protein-coding genes were annotated in this study. We found some CDSs and CNSs with relatively high nucleotide diversity (**Table S2**; **Figure S3**), which was in line with previous research (Xue et al., 2019; Liu et al., 2020; Wang et al., 2021). Analyses of cp genes and genomes have largely contributed to resolving portions of the plant tree of life (Ravi et al., 2007). Various genes and IGRs have been identified as evolutionarily significant markers, which have been widely used for phylogenetic analyses (Ravi et al., 2007). Intergenic spacer regions were proposed to be the best barcoding candidates (Ravi et al., 2007), which was also confirmed in this study, and we further found that the *Pi* of the IGRs was higher than that of the CDSs (**Table S2**; **Figure S3**). Nevertheless, both the IGRs and CDSs can serve as useful molecular markers. Some genes (*matK*, *rps16*, *rbcL*, *rpl16*, *ndhA, ndhF*, and *ycf1*) and IGRs (*rps16*-*trnQ*, *petN*-*psbM*, *rps15*-*ycf1*, *trn*L-*trnF*) exhibited high nucleotide diversity (**Table S2**), which have also been used for phylogenetic and phylogeography analyses (Ravi et al., 2007; Chavez et al., 2016; Uchoi et al., 2016; Yang et al., 2016; Khan et al., 2018).

### 4.2 Evolutionary and phylogenetic analysis

Repeats play an important role in genome rearrangement, which can increase the probability of replication fork stagnation, causing an error to recruit persistently specific sequence regions over evolutionary time scales (Mcdonald et al., 2011). Repetitive sequences may facilitate intermolecular recombination and enhance plastome diversity, as an abundance of sequence repeats results in genome regions with increased sequence diversity in prokaryotes and eukaryotes (Mcdonald et al., 2011; Liu et al., 2020). We also found abundant repeats in the *Cerasus* genome, including dispersed, palindromic, and tandem repeats, along with SSRs (**Figure 4A**; **Table S4A**). More abundant SSRs were detected in IGRs for most species, except for *P. tianshanica* (**Figure 4A**; **Table S4A**) and other *Prunus* species (Wang et al., 2021; Zhang et al., 2021). Combined with the visualization of aligned genome sequences, CNSs showed more significant divergence (**Figure 2**). This suggested that repetitive CNSs might be the main force promoting cp genome rearrangement in *Prunus* species (Wang et al., 2021). Furthermore, given the characteristics of maternal inheritance, conservation, and simple structure of the cp genome, cp microsatellites with a high degree of polymorphism can serve as useful molecular markers to identify genetic relationships, population genetic structure, and phylogeography patterns at the inter- and intrapopulation levels (Decroocq et al., 2004; Wang et al., 2021). We only detected mono-, di-, and tri-nucleotide repeats in the *Cerasus* cp genomes (**Figure 4**; **Table S4**) with a greater content of A/T repeats than of G/C repeats, similar to the results of other studies in the genus *Prunus* (Xue et al., 2019; Zhang et al., 2021). The SSRs identified in this study provide useful information for developing genetic markers to further study the population genetics, evolution, and breeding of the subgenus *Cerasus*, as well as for the identification and conservation of *Cerasus* species. Repetitive sequences are also essential for research on indels and substitutions (Wang et al., 2021), which are highly abundant in the plastome of *Cerasus* (**Table S3**) and other members of the family Rosaceae (Zhang et al., 2021).

Codon usage and synonymous/nonsynonymous substitutions play an important role in cp plastome evolution (Ivanova et al., 2017; Huang et al., 2021). Mutation is one of the most essential factors affecting codon usage, thus influencing the evolutionary course (Ivanova et al., 2017; Wang et al., 2021). Moreover, codon-choice patterns are considered to be highly conserved during the evolution process (Ikemura, 1985). An RSCU value > 1, < 1, or = 1 indicates preference, low usage, and no preference for a codon, respectively (Sharp and Li, 1987). We found biased codon usage in the *Cerasus* cp genome, with 19 amino acids having an RSCU value >1 (**Figure S5**). The codon profile showed strong bias toward the use of A/T in the third-base position, which appears to be a general phenomenon (Murray et al., 1989). Leucine and isoleucine appeared the most frequently and were biased toward UUA and AUU, respectively (**Figures S5**, **5**), whereas cysteine was the least frequently detected, with the third base also biased toward T (UGU) in codon usage (**Figures S5**, **5**). Among the three stop codons, there was clear usage bias toward UAA (RSCU > 1.00) (**Figure S5**). These results are largely consistent with reports of the cp genomes in *P. zhengheensis* (Huang et al., 2021; Wang et al., 2021) and other species (Alzahrani et al., 2020; Liu et al., 2020). Two amino acids, methionine (AUG) and tryptophan (UGG), showed no codon usage bias (RSCU = 1.00) (**Figure S5**). In other words, all amino acids are encoded by 2–6 synonymous codons with the exception of methionine and tryptophan (Murray et al., 1989).

The Ka and Ks nucleotide substitution rates as well as the Ka/Ks ratio are widely used to evaluate the sequence divergence and purifying selection in protein-coding genes. In most genes, with the exception of very rapidly evolving genes, Ka nucleotide substitutions occur less frequently than Ks owing to the action of purifying selection (Ivanova et al., 2017). Ks nucleotide substitutions generally occur more frequently than Ka substitutions (Liu et al., 2017), which was also detected for *Cerasus* in this study. Among the changed genes, almost all Ka/Ks ratios were less than 1.0 (**Tables S5**, **S6**), providing evidence for purifying selection on the protein-coding genes of the cp genome in the genus *Prunus* and family Rosaceae. According to the Ka/Ks values, we found that *rpoA*, *atpA, petB*, *cemA*, and *rpoC1* exhibited strong purifying selection. The *ccsA* was under neutral selection, whereas *ycf2* genes showed a signature of possible positive selection during the course of *Cerasus* evolution (**Table S6**), owing to an inserted fragment in the middle of the gene (**Figure S2A**), in line with the Ka/Ks analyses. However, these patterns are in contrast to previous research on *Cerasus* (Zhang et al., 2021), in which the *matK* and *rpoC2* genes both showed signatures of positive selection, along with other genes such as *ndhF*, *atpA*, and *psaA*. Under conditions of extreme temperature and changing light intensity, the *ndhF* gene can balance the redox levels to maintain or enhance photosynthetic performance (Martin et al., 2009; Zhang et al., 2021). In addition, Zhang et al. (2021) detected strong signatures of positive selection in several genes of Rosaceae, including *rpoA*, *rps16, rps18, psaA, psbL, rbcL, ndhD, ndhF, accD, ycf1*, and *ycf2*. In particular, the *rbcL* gene encodes the large subunit of RuBisCO, which is one of the most useful enzymes for studying plant evolution, serving as a model protein in several studies owing to its response to environmental pressure and climate shifts (Hermida-Carrera et al., 2017; Zhang et al., 2021). In addition to *rbcL*, *ndhF*, *ycf1*, and *rps18* had high *Pi* values in this study, which can help Rosaceae woody fruit trees efficiently capture light energy to obtain sufficient nutrition for growth and development as an adaptation under extreme and variable environmental conditions (**Table S2**). Hence, it is necessary to further study the patterns of synonymous and non-synonymous substitutions among *Cerasus* species, which can provide new insight into the evolution of Rosaceae.

The subgenus *Cerasus* can be classified in two sections of true cherry (*Cerasus sensu stricto*) and dwarf cherry (*Microcerasus*), according to Yü et al. (1986). The genetic evolution of *Cerasus* species has been a long-standing open research question (Liang et al., 2018; Zhang et al., 2021). Given its maternal inheritance, the cp genome has been widely used for species classification and evolutionary analyses (Wang et al., 2022). Therefore, we reconstructed the *Cerasus* phylogenetic tree based on the complete cp genome (**Figure 6**). In line with the classification proposed by Yü et al. (1986) and others, true cherry species were classified in a single group as clade I (**Figure 6**). Surprisingly, *P. tianshanica*, as a *Microcerasus* species, also clustered in the true cherry group and formed a clade with *P. cerasus*, *P. fruticosa*, *P. cerasus* × *P. canescens* ‘Gisela 6’, and *P. avium*. Moreover, *P. mahaleb* in clade I was separated from other true cherry species, which is in line with previous reports (Chin et al., 2014; Zhang et al., 2021). Further subdivisions of true cherry match the existing classification (Yü et al., 1986; Webster and Looney, 1996) proposing *P. mahaleb* or *P. emarginata*, *P. pennsylvanica*, and *P. prunifolia* as a separate group, named section *Mahaleb* Focke (**Table S7**). However, in the present study, *P. emarginata* was not grouped with *P. mahaleb* (**Figure 6**). In addition, we found that some true cherry species did not follow the grouping of existing classifications (Yü et al., 1986; Webster and Looney, 1996) or varied to a certain extent at the section level, such as *P. tianshanica*, *P. serrula*, and *P. serrulata* (**Figure 6**; **Table S7**). *Microcerasus* also showed a significant difference from other taxa (**Table S7**) (Yü et al., 1986; Webster and Looney, 1996), which were grouped with *Amygdalus, Armeniaca*, and *Prunus* as a subclade in this study (**Figure 6**). These relationships were also reported in previous studies (Chen et al., 2018; Zhang et al., 2021; Li et al., 2022). *Microcerasus* showed close evolutionary relationships to *Amygdalus* (*P. tenella*, *P. pedunculata*, and *P. triloba*) and *Armeniaca* (*P. mume*, *P. mandshurica*, and *P. armeniaca*) species. Specifically, *P. japonica* and *P. tomentosa* were the closest relatives to *Amygdalus* (*P. triloba* and *P. pedunculata*). Previous studies also revealed that *P. pesica* in *Amygdalus* was closely related to *Microcerasus* (Chen et al., 2018; Wang et al., 2020; Zhang et al., 2021). Hence, during the evolution process, true cherry formed a distinct group, while *Microcerasus* remained genetically closer to *Amygdalus*, *Armeniaca*, and *Prunus* (*sensu stricto*) than to true cherry. These results can be supported by multi-cp genome comparative (Zhang et al., 2021) and whole-genome analyses (Wang et al., 2020). Nevertheless, further breakdown of *Cerasus* (*sensu stricto*) should not be ignored based on these results, which contrast with the existing classification criteria (Yü et al., 1986; Webster and Looney, 1996). Accordingly, further study of the taxa of the subgenus *Cerasus*, especially *Microcerasus*, is necessary to enable breeding novel cherry cultivars and to gain a better understanding of the evolution of *Prunus sensu lato* specifically and Rosaceae plants more broadly.

## 5 Conclusion

Comparative analysis of the cp genome is a key approach to study the molecular evolution and reconstruct the phylogenetic tree of *Cerasus* species. The present analysis of the cp genomes of 11 *Cerasus* species showed that IR regions are more strongly conserved than the LSC and SSC regions, whereas the non-coding sequences showed more significant divergence than the coding regions. The contraction/expansion of *rps19* and *ndhF* at the IR boundaries were the main contributors to the observed variation among *Cerasus* cp genomes, as well as variation in *ycf1* and *trnH*. We identified 26 genes and IGRs with variations that can be used as potential molecular markers and candidate DNA barcodes for studying the phylogeny and phylogeography of cherry species. We further provided important evidence that *P. mahaleb* forms a unique clade among true cherry (*Cerasus sensu stricto*) due to plastid genome rearrangement. *Microcerasus* was found to be genetically closer to *Amygdalus*, *Armeniaca*, and *Prunus* (*sensu stricto*) than to true cherry species. Moreover, *P. tianshanica* emerged as a noteworthy species given its close relationship to *P. avium*. Overall, these findings provide new insight and resources to breed novel cultivated sweet cherry and cherry rootstock in the future.

## Data availability statement

The datasets presented in this study can be found in online repositories. The names of the repository/repositories and accession number(s) can be found in the article/**Supplementary Material**.

## Author contributions

TW and Y-LC designed the research. TW performed the experiments and analyzed the data. Y-LC identified the plant materials and revised the manuscript. B-XQ, JZ, and K-SS revised the manuscript. FA assembled the sequences. TW, B-XQ, JZ, L-YP, and FA annotated the plastomes. TW, K-SS, L-YP, X-SH, TL, and P-KL collected the plant materials. FA and L-YP provided analysis support. All authors contributed to the article and approved the submitted version.
