# Supplementary material for: Evolutionary and phylogenetic analyses of 11 Cerasus species based on the complete chloroplast genome
Source: Front Plant Sci. 2023 Mar 3;14:1070600. doi: 10.3389/fpls.2023.1070600 (PMC10022824; doi:10.3389/fpls.2023.1070600)
Supplement: Supplementary file 1 [file DataSheet_1.docx]

Supplementary Figures


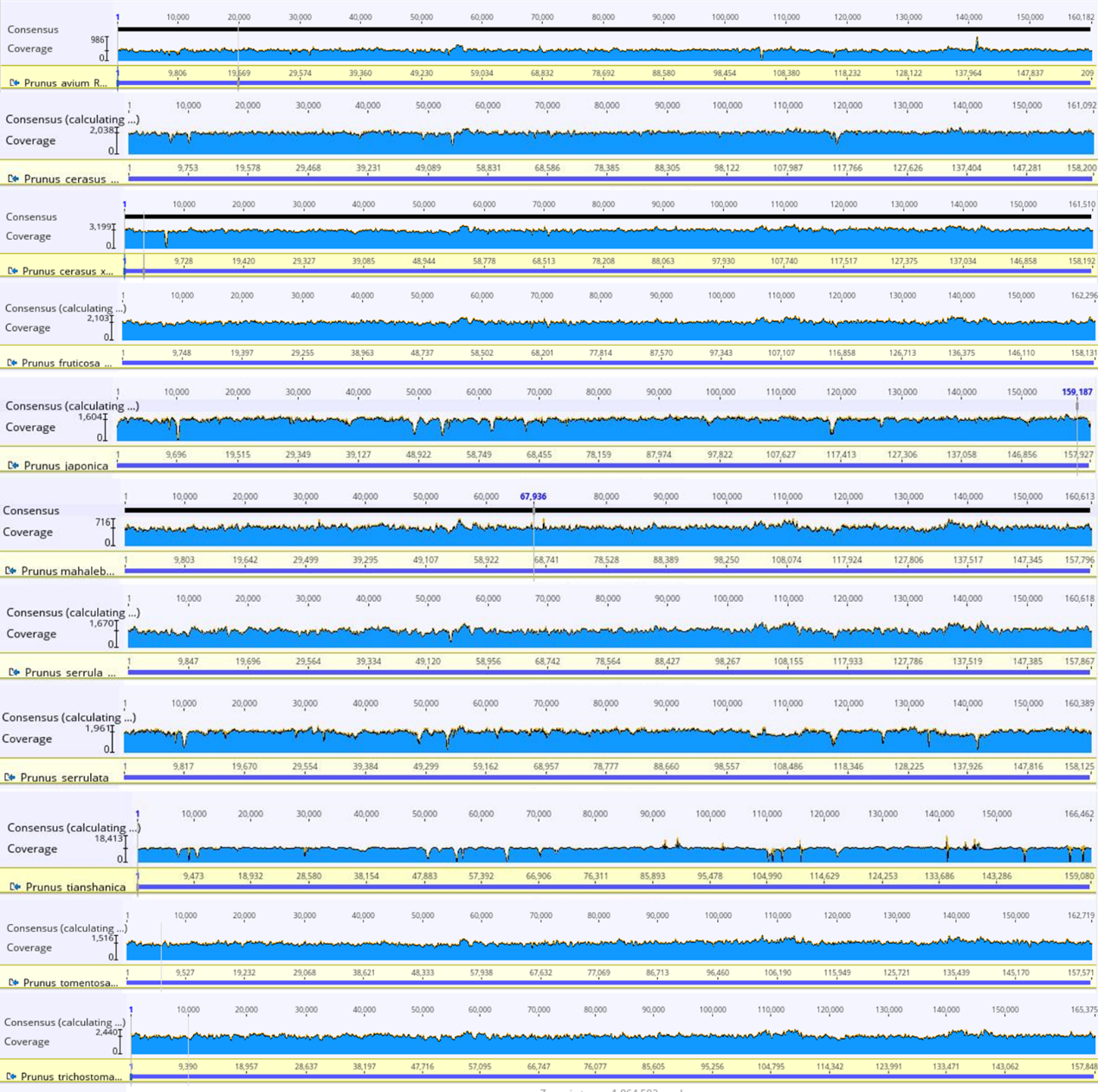


**Figure S1** Clean reads mapped to the assembled plastome of 11 *Cerasus* species.


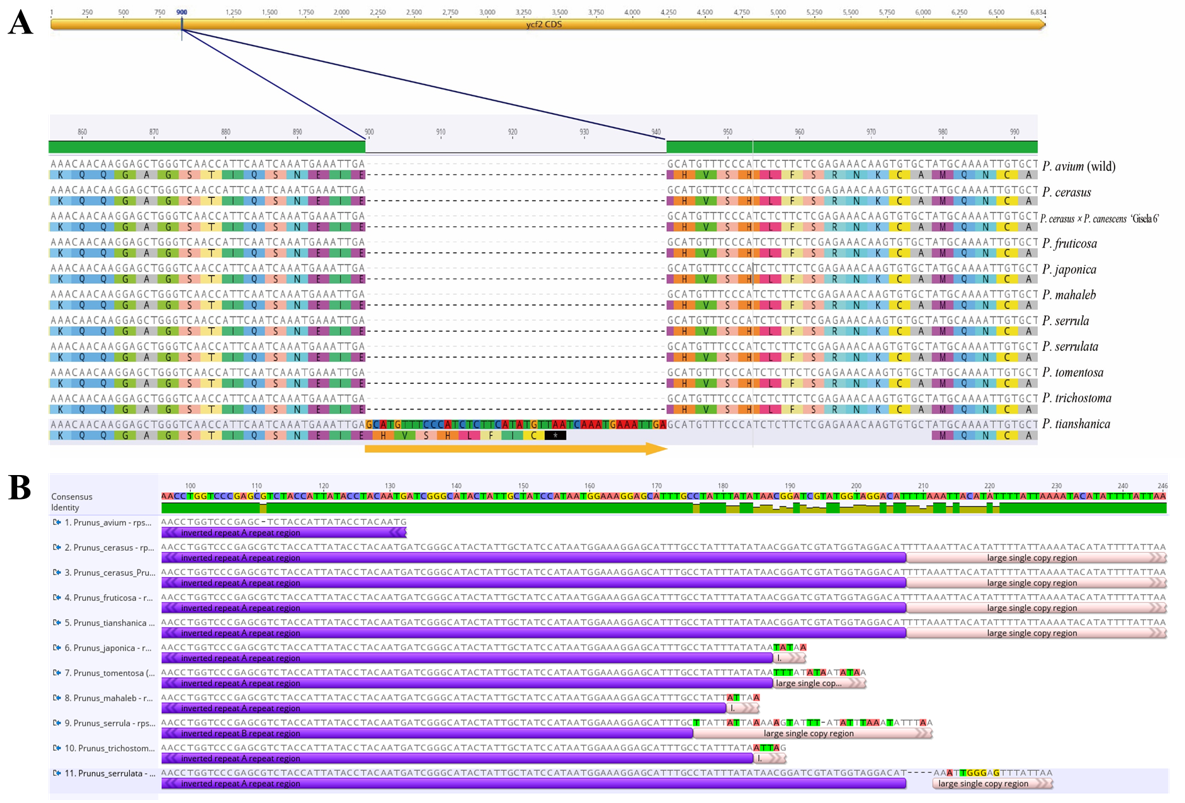


**Figure S2** Sequence alignment of the *ycf2*_IRA gene and *rps19* gene. (**A**) Comparison of the *ycf2*_IRA gene in 11 *Cerasus* species. The *ycf2* gene in *Prunus tianshanica* is divided two fragment by one inserted sequence labeled with the yellow arrow. (**B**) Sequence alignment of the *rps19* gene on the boundary of the IRA and LSC regions.


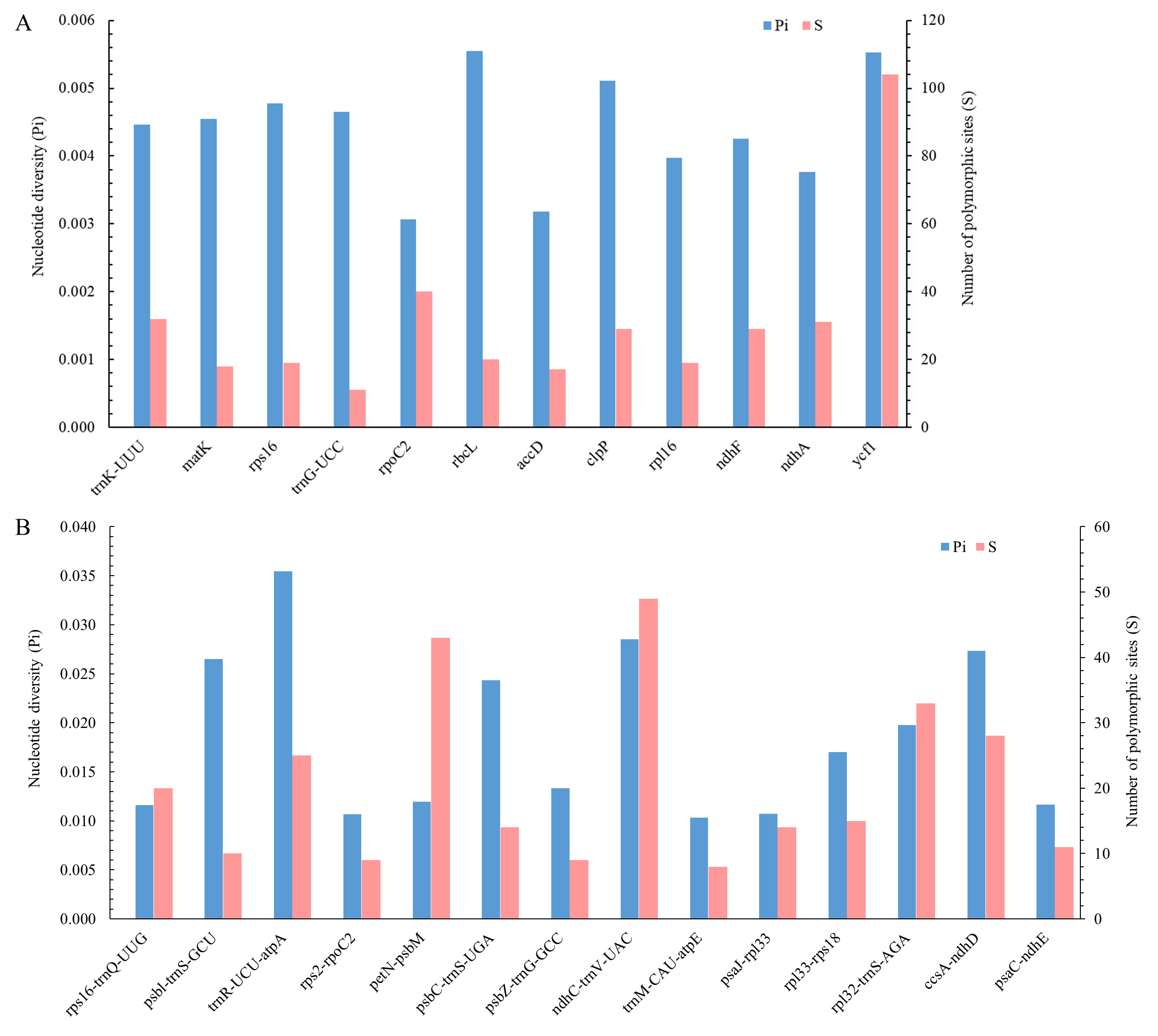


**Figure S3** Plastid genes with *Pi* > 0.003 and intergenic regions with *Pi* > 0.01 and *S* >10 of 12 *Cerasus* species plastomes computed by DnaSP v.6, including the 11 species analyzed in this study and an additional species, *Prunus pseudocerasus* (NC030599), downloaded from NCBI.


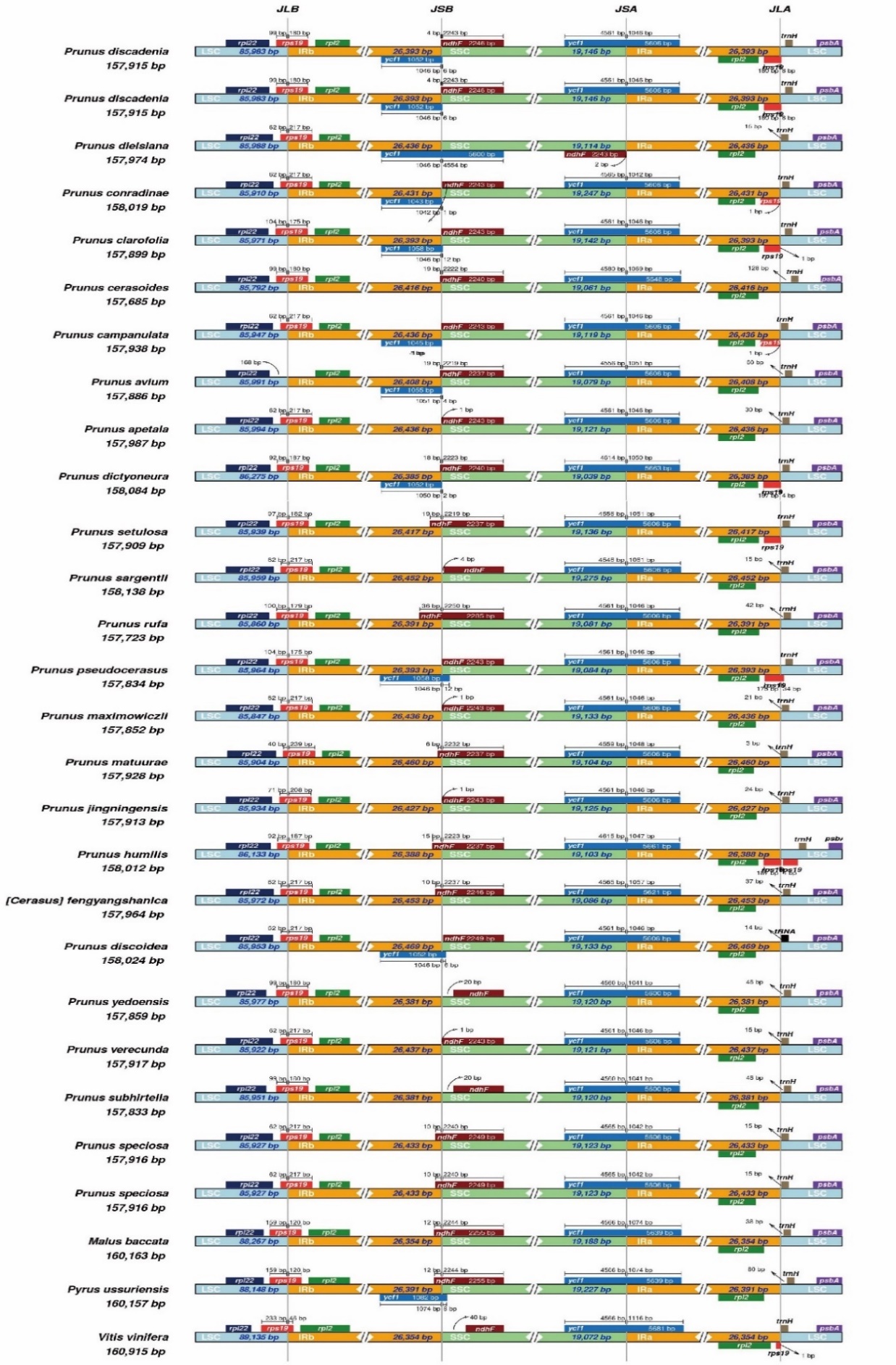
**Figure S4** Comparison of the IR/LSC and IR/SSC boundaries among 28 chloroplast genomes of 25 *Cerasus* species and three outgroup species downloaded from NCBI.


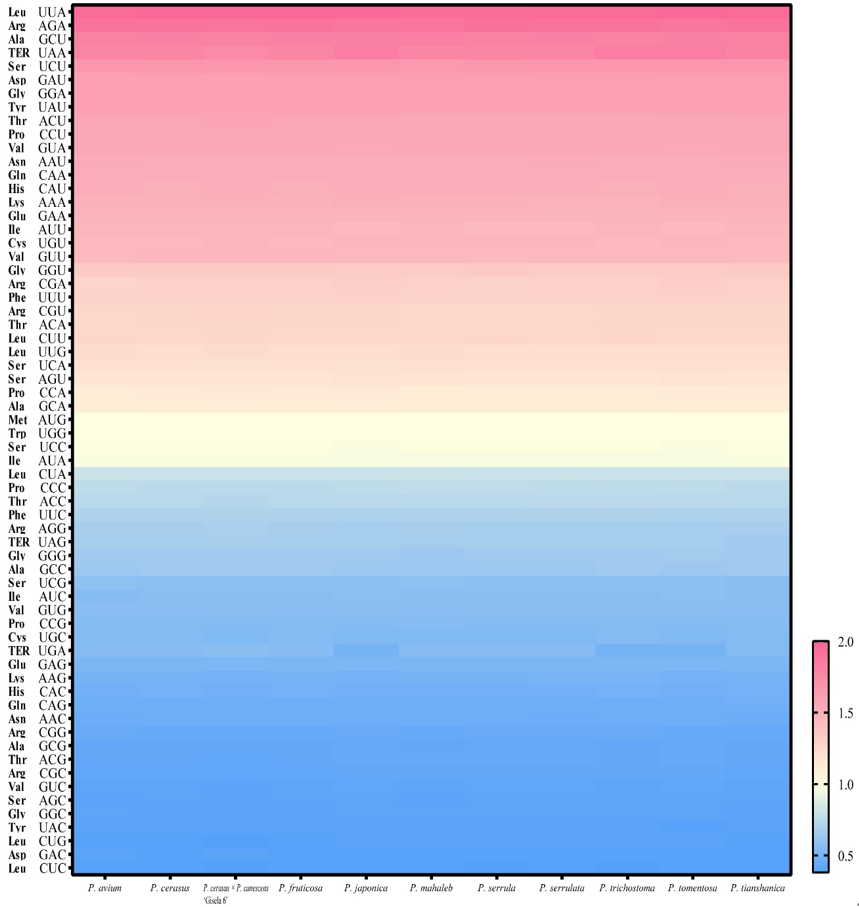


**Figure S5** Visualization of the relative synonymous codon usage (RSCU) patterns in 11 *Cerasus* species. The color scale indicates the magnitude of overall RSCU values: the reddest codons are the most preferred (A/T-ending codons) and the bluest codons are the least preferred (C/G-ending codons).


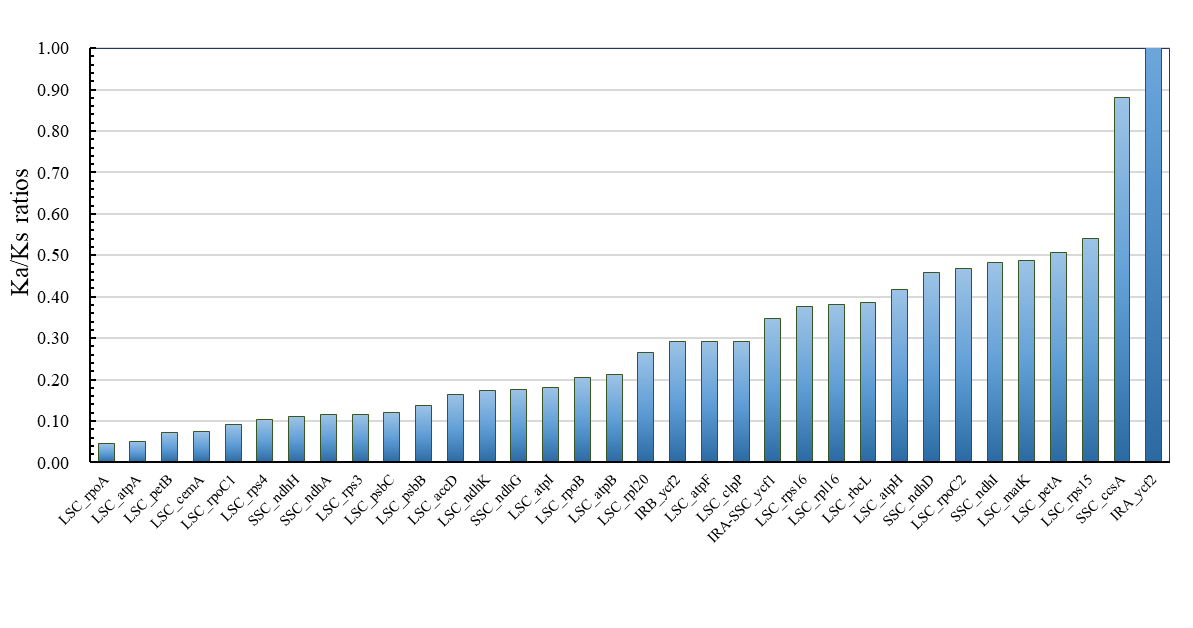


**Figure S6** Average Ka/Ks ratio for 34 protein-coding genes in the 11 *Cerasus* chloroplast genomes.


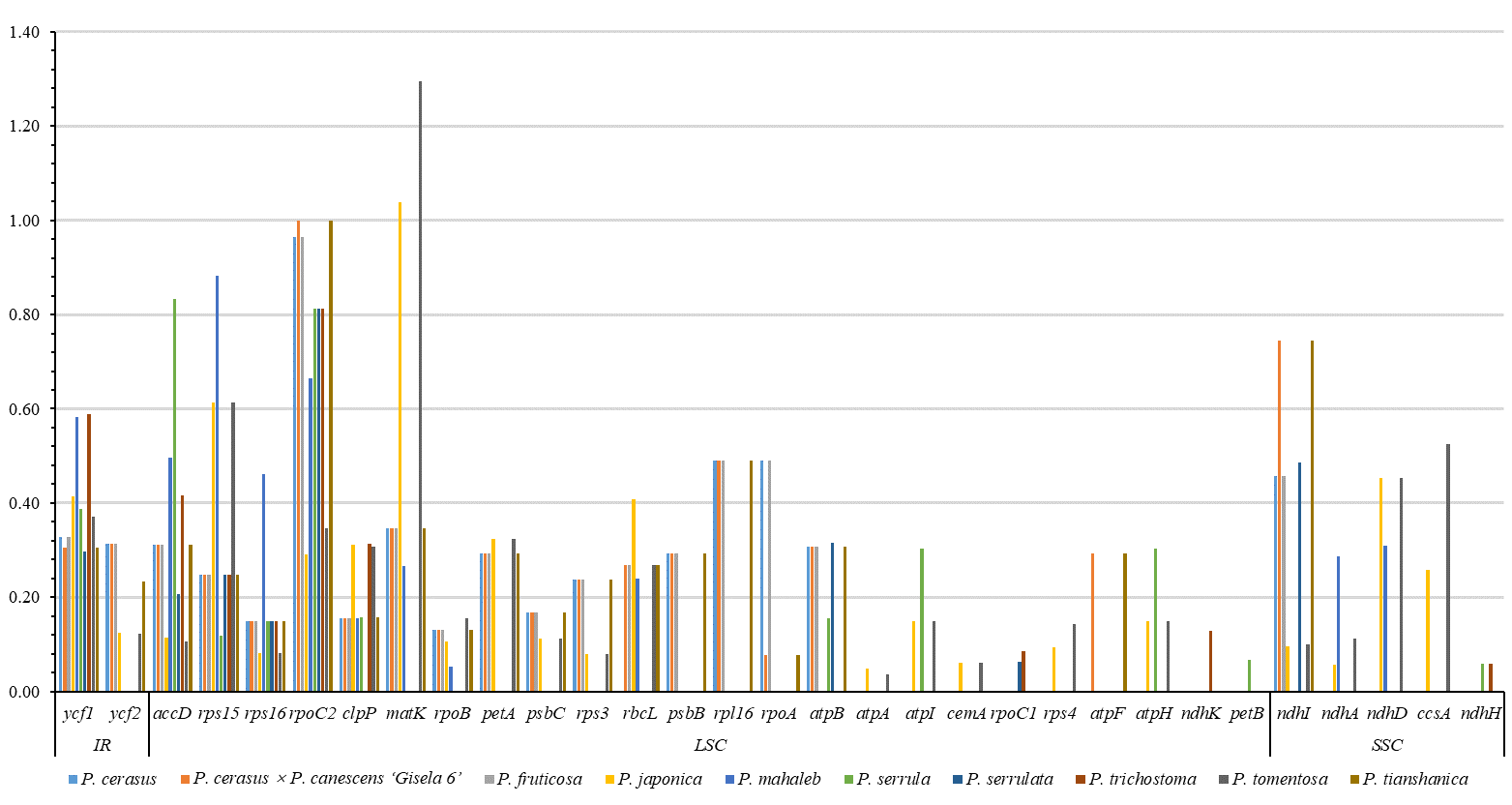


**Figure S7** Ka/Ks ratios of 31 protein-coding genes of chloroplast genomes of 10 *Cerasus* species in comparison with *P. avium*.

**
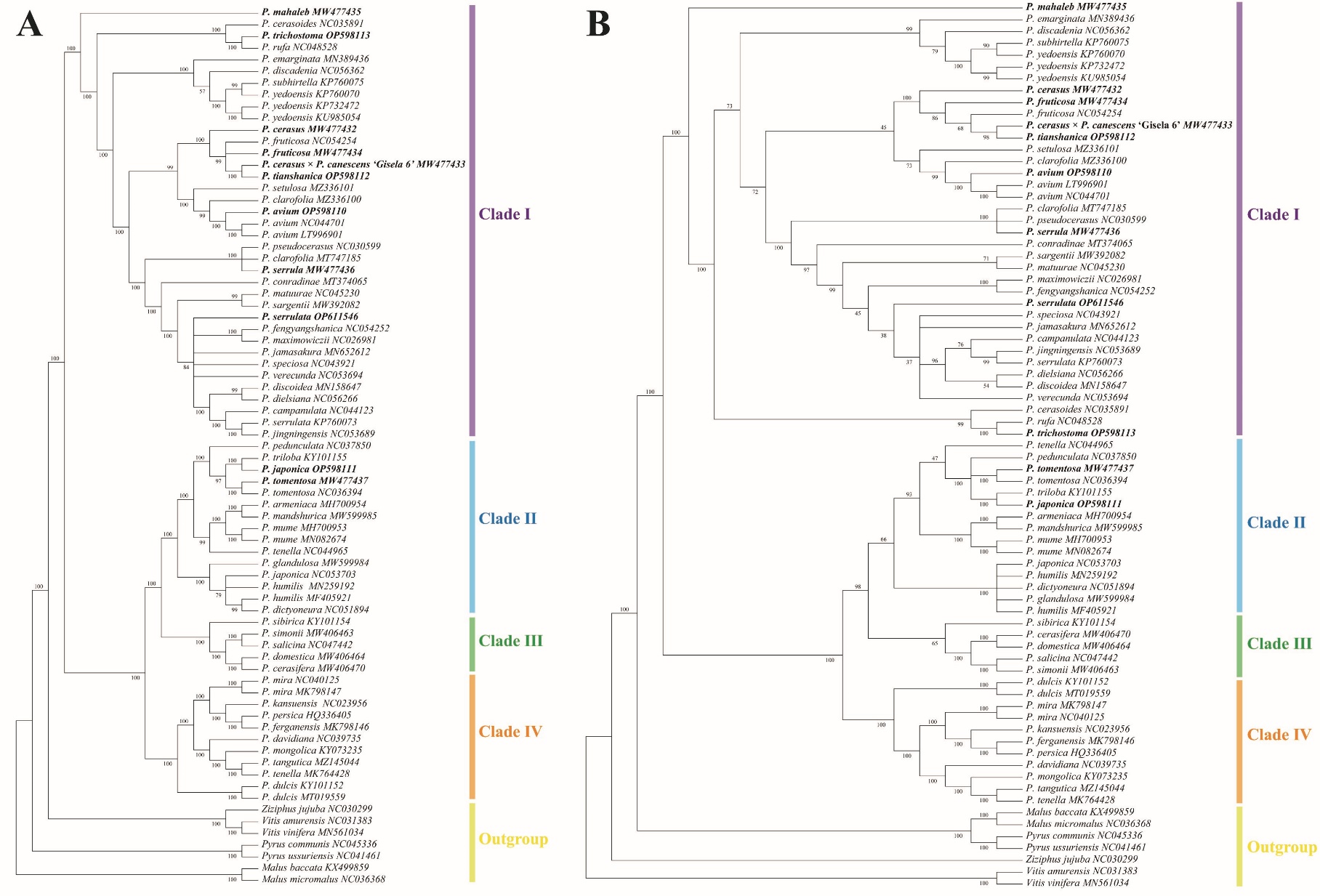
**

**Figure S8** Phylogenetic tree reconstruction of 45 *Cerasus* species and 23 other Prunoideae species based on large single-copy region sequences. (**A)** Phylogenetic tree reconstruction using the program Mrbayes of Geneious Prime v2022.0.2. Numbers above the lines represent the Bayesian inference posterior probability (percent). (**B)** Phylogenetic tree reconstruction using Maximum likelihood method of MEGA v11. Numbers below the lines represent the bootstrap support values.

**
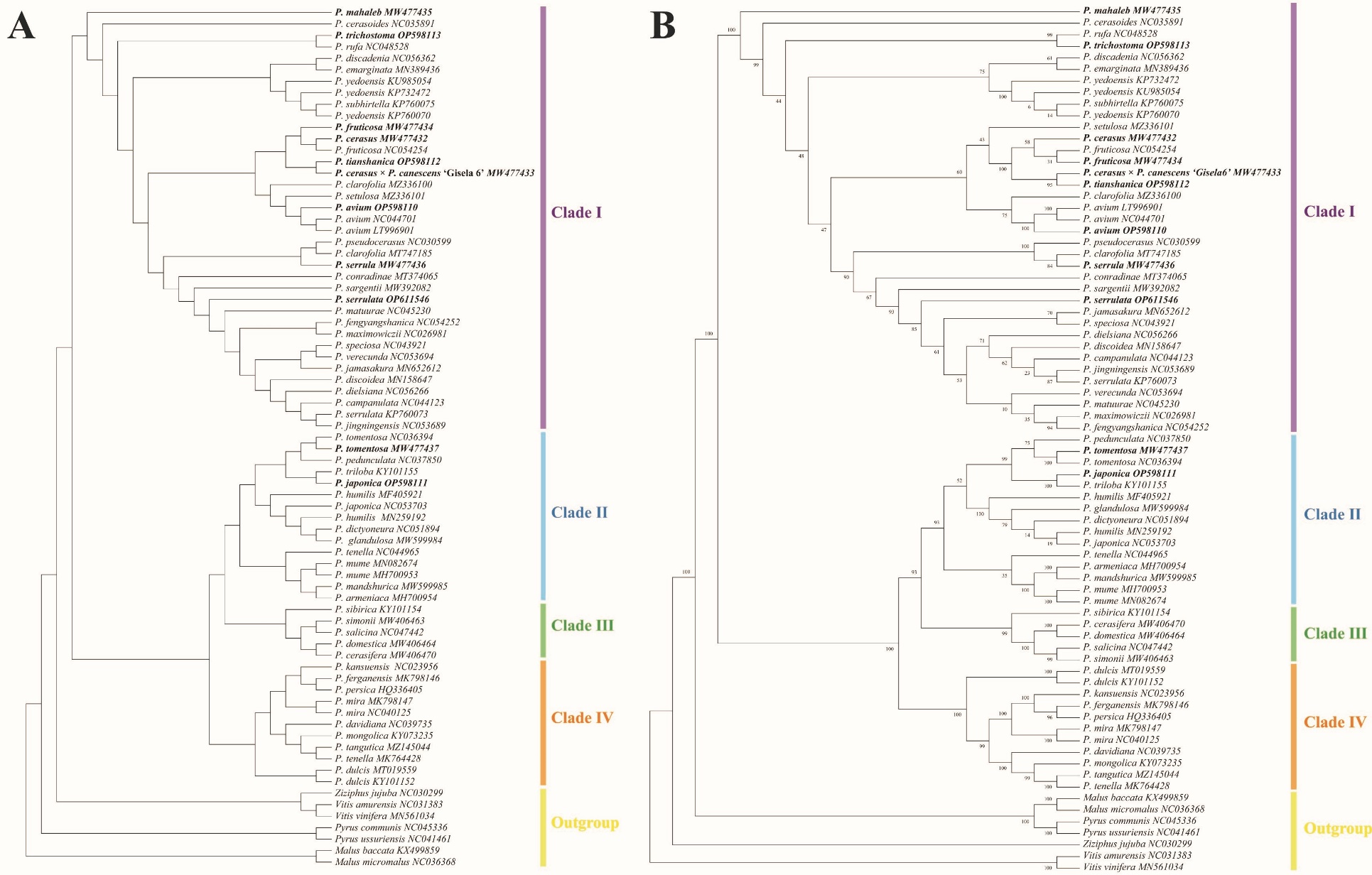
**

**Figure S9** Phylogenetic tree reconstruction of 45 *Cerasus* species and 23 other Prunoideae species based on small single-copy region sequences. (**A)** Phylogenetic tree reconstruction using the program Mrbayes of Geneious Prime v2022.0.2. Numbers above the lines represent the Bayesian inference posterior probability (percent). (**B)** Phylogenetic tree reconstruction using Maximum likelihood method of MEGA v11. Numbers below the lines represent the bootstrap support values.

**
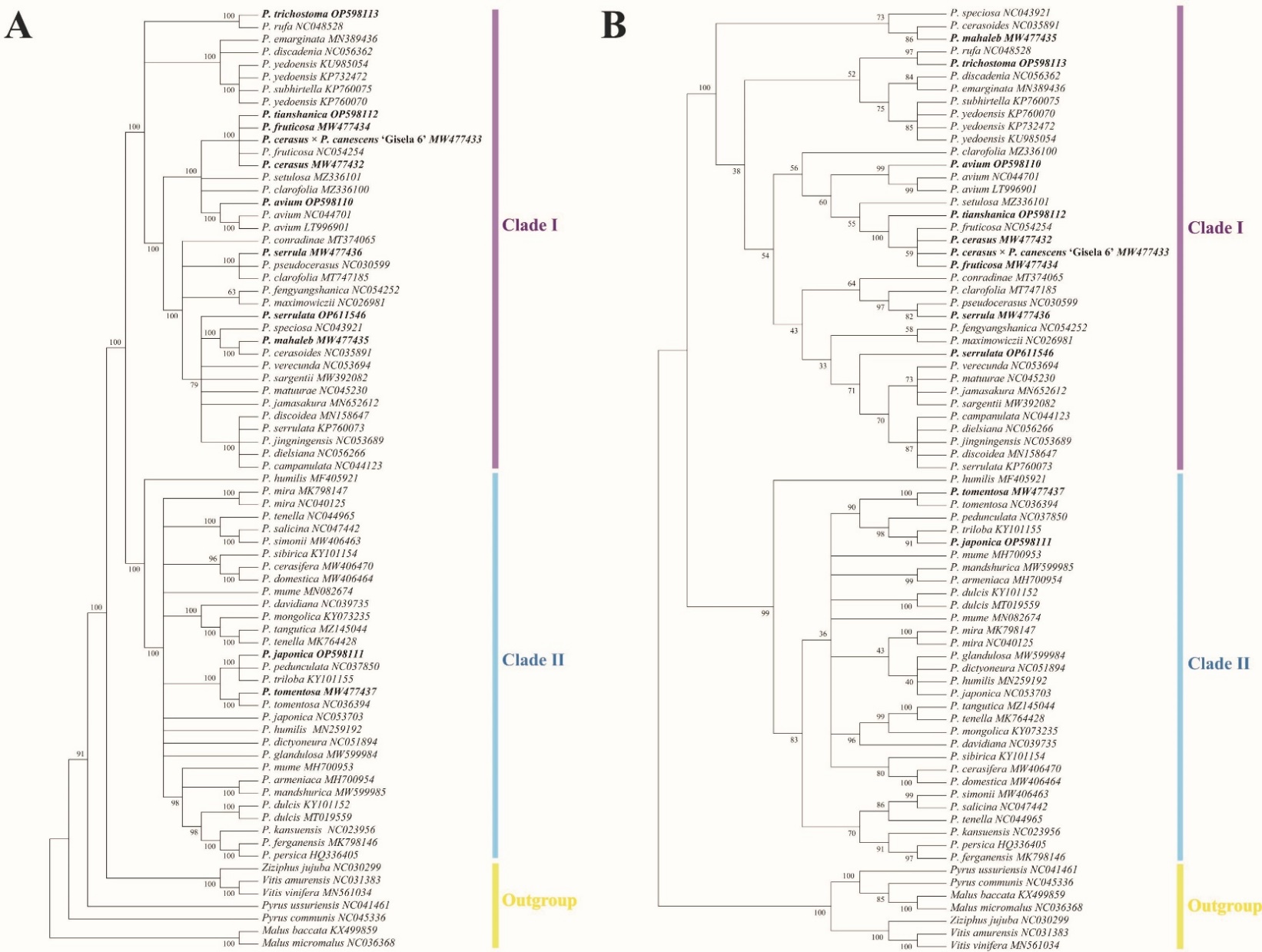
**

**Figure S10** Phylogenetic tree reconstruction of 45 *Cerasus* species and 23 other Prunoideae species based on two copies of an inverted repeat sequences. (**A)** Phylogenetic tree reconstruction using the program Mrbayes of Geneious Prime v2022.0.2. Numbers above the lines represent the Bayesian inference posterior probability (percent). (**B)** Phylogenetic tree reconstruction using Maximum likelihood method of MEGA v11. Numbers below the lines represent the bootstrap support values.

**
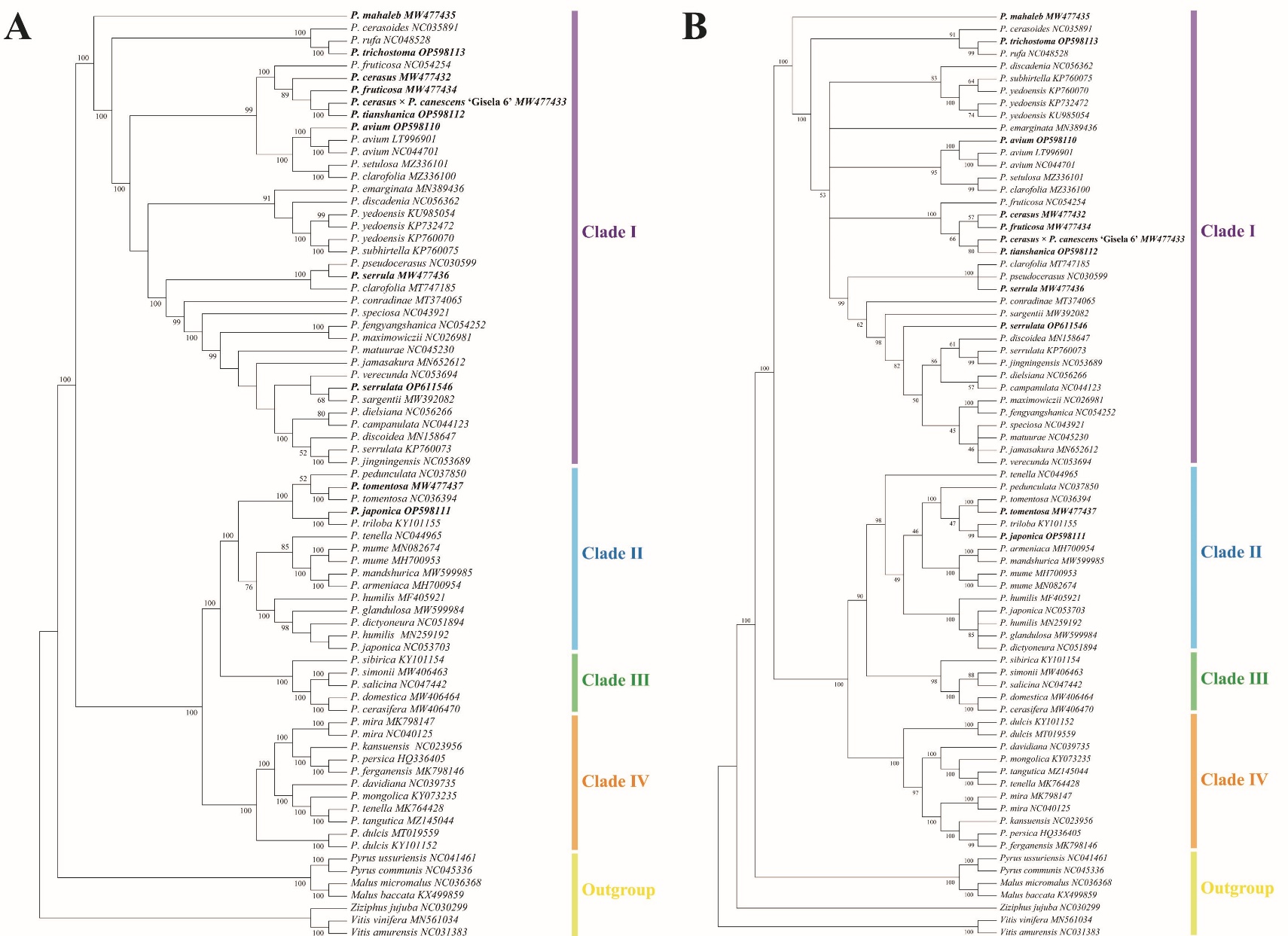
**

**Figure S11** Phylogenetic tree reconstruction of 45 *Cerasus* species and 23 other Prunoideae species based on the common protein-coding sequences. (**A)** Phylogenetic tree reconstruction using the program Mrbayes of Geneious Prime v2022.0.2. Numbers above the lines represent the Bayesian inference posterior probability (percent). (**B)** Phylogenetic tree reconstruction using Maximum likelihood method of MEGA v11. Numbers below the lines represent the bootstrap support values.

**
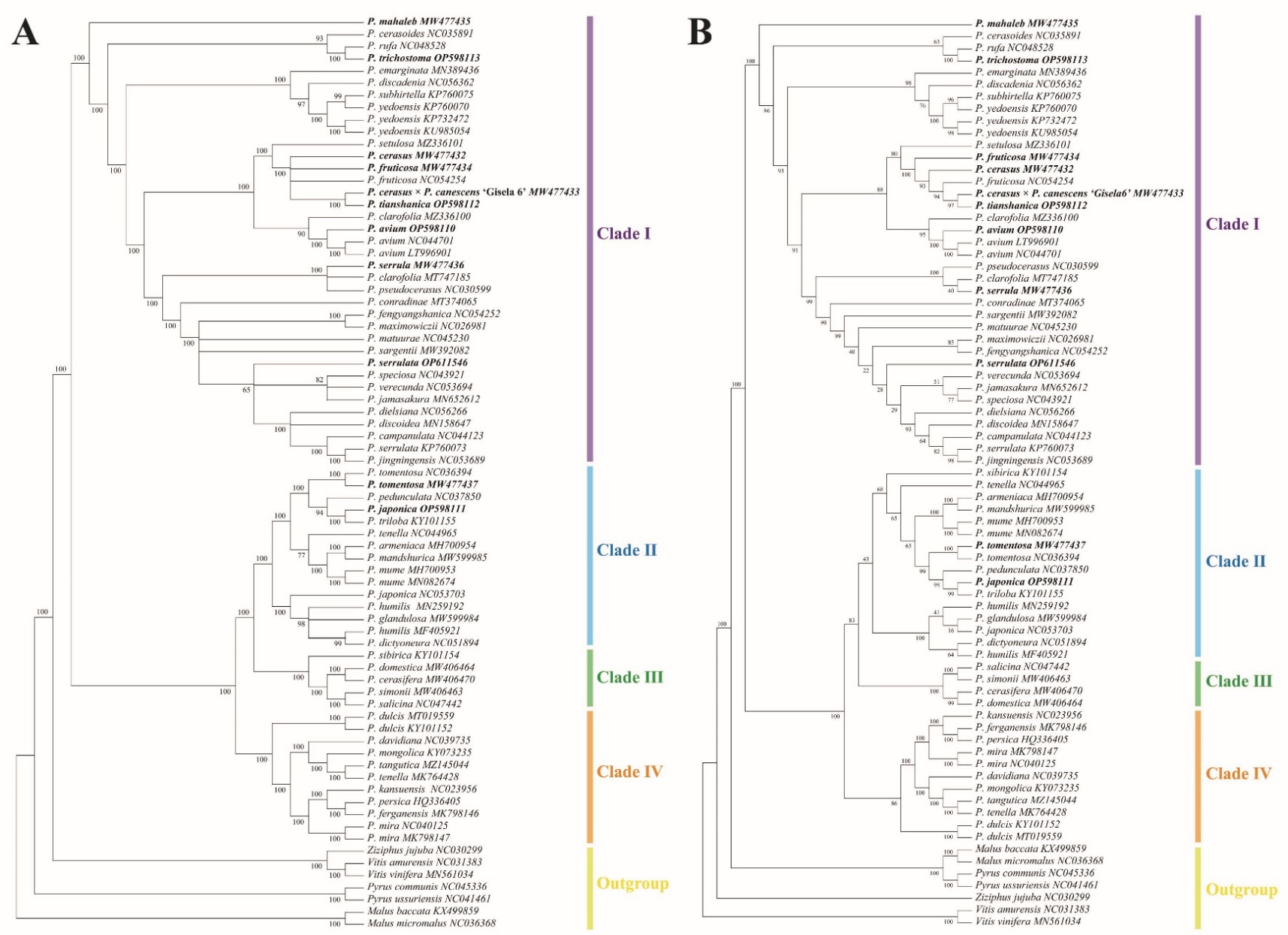
**

**Figure S12** Phylogenetic tree reconstruction of 45 *Cerasus* species and 23 other Prunoideae species based on the common intergenic regions sequences. (**A)** Phylogenetic tree reconstruction using the program Mrbayes of Geneious Prime v2022.0.2. Numbers above the lines represent the Bayesian inference posterior probability (percent). (**B)** Phylogenetic tree reconstruction using Maximum likelihood method of MEGA v11. Numbers below the lines represent the bootstrap support values.
